# Exploration and validation of key genes associated with early lymph node metastasis in thyroid carcinoma using weighted gene co-expression network analysis and machine learning

**DOI:** 10.3389/fendo.2023.1247709

**Published:** 2023-12-08

**Authors:** Yanyan Liu, Zhenglang Yin, Yao Wang, Haohao Chen

**Affiliations:** ^1^ Department of General Surgery, The Third Affiliated Hospital of Anhui Medical University (The First People’s Hospital of Hefei), Hefei, Anhui, China; ^2^ Digestive Endoscopy Department, Jiangsu Province Hospital, The First Affiliated Hospital with Nanjing Medical University, Nanjing, Jiangsu, China

**Keywords:** thyroid cancer, bioinformatics analysis, The Cancer Genome Atlas, nomogram, machine learning

## Abstract

**Background:**

Thyroid carcinoma (THCA), the most common endocrine neoplasm, typically exhibits an indolent behavior. However, in some instances, lymph node metastasis (LNM) may occur in the early stages, with the underlying mechanisms not yet fully understood.

**Materials and methods:**

LNM potential was defined as the tumor’s capability to metastasize to lymph nodes at an early stage, even when the tumor volume is small. We performed differential expression analysis using the ‘Limma’ R package and conducted enrichment analyses using the Metascape tool. Co-expression networks were established using the ‘WGCNA’ R package, with the soft threshold power determined by the ‘pickSoftThreshold’ algorithm. For unsupervised clustering, we utilized the ‘ConsensusCluster Plus’ R package. To determine the topological features and degree centralities of each node (protein) within the Protein-Protein Interaction (PPI) network, we used the CytoNCA plugin integrated with the Cytoscape tool. Immune cell infiltration was assessed using the Immune Cell Abundance Identifier (ImmuCellAI) database. We applied the Least Absolute Shrinkage and Selection Operator (LASSO), Support Vector Machine (SVM), and Random Forest (RF) algorithms individually, with the ‘glmnet,’ ‘e1071,’ and ‘randomForest’ R packages, respectively. Ridge regression was performed using the ‘oncoPredict’ algorithm, and all the predictions were based on data from the Genomics of Drug Sensitivity in Cancer (GDSC) database. To ascertain the protein expression levels and subcellular localization of genes, we consulted the Human Protein Atlas (HPA) database. Molecular docking was carried out using the mcule 1-click Docking server online. Experimental validation of gene and protein expression levels was conducted through Real-Time Quantitative PCR (RT-qPCR) and immunohistochemistry (IHC) assays.

**Results:**

Through WGCNA and PPI network analysis, we identified twelve hub genes as the most relevant to LNM potential from these two modules. These 12 hub genes displayed differential expression in THCA and exhibited significant correlations with the downregulation of neutrophil infiltration, as well as the upregulation of dendritic cell and macrophage infiltration, along with activation of the EMT pathway in THCA. We propose a novel molecular classification approach and provide an online web-based nomogram for evaluating the LNM potential of THCA (http://www.empowerstats.net/pmodel/?m=17617_LNM). Machine learning algorithms have identified ERBB3 as the most critical gene associated with LNM potential in THCA. ERBB3 exhibits high expression in patients with THCA who have experienced LNM or have advanced-stage disease. The differential methylation levels partially explain this differential expression of ERBB3. ROC analysis has identified ERBB3 as a diagnostic marker for THCA (AUC=0.89), THCA with high LNM potential (AUC=0.75), and lymph nodes with tumor metastasis (AUC=0.86). We have presented a comprehensive review of endocrine disruptor chemical (EDC) exposures, environmental toxins, and pharmacological agents that may potentially impact LNM potential. Molecular docking revealed a docking score of -10.1 kcal/mol for Lapatinib and ERBB3, indicating a strong binding affinity.

**Conclusion:**

In conclusion, our study, utilizing bioinformatics analysis techniques, identified gene modules and hub genes influencing LNM potential in THCA patients. ERBB3 was identified as a key gene with therapeutic implications. We have also developed a novel molecular classification approach and a user-friendly web-based nomogram tool for assessing LNM potential. These findings pave the way for investigations into the mechanisms underlying differences in LNM potential and provide guidance for personalized clinical treatment plans.

## Introduction

The continuous advancement in detection technology has resulted in an ongoing rise in the rate of thyroid carcinoma (THCA) detection. Compared to other types of endocrine malignancies, THCA holds the highest prevalence, experiencing an annual increase in its incidence (1). Surgical resection is the primary treatment modality for THCA. Post-surgery, the decision to perform neck lymph node dissection or radioactive iodine therapy should be based on the patient’s condition and pathological type. Additional treatment modalities include radioisotope therapy, endocrine inhibition therapy, and external beam radiation therapy (mainly used for anaplastic thyroid cancer), among others. Despite typically displaying an indolent nature and promising overall prognosis, THCA has a significant potential to exhibit an invasive phenotype and in some cases may metastasize (2). Recent reports indicate an approximate 38.5%~58.8% rate of lymph node metastasis (LNM) in THCA (3). Moreover, cervical LNM may occur at the early stages of disease progression (4). The presence of LNM serves as a key indicator for prognosis and treatment options in individuals afflicted with THCA (5). In cases where LNM is detected, a comprehensive approach incorporating radical surgery with lymph node dissection is deemed necessary (6). Furthermore, the implementation of iodine-131 treatment may also be considered based on specific indications (7). LNM constitutes an important prognostic determinant, exhibiting a close association with both tumor recurrence and unfavorable prognostic outcomes among individuals afflicted with THCA (8). Additionally, performing neck lymph node dissection due to suspected cervical lymph node metastasis can potentially lead to damage to glands and nerves, such as the internal jugular vein, submandibular gland, brachial plexus, and accessory nerve. This can also result in adverse postoperative outcomes for the patients (9). Hence, gaining clarity regarding the occurrence or inclination towards lymph node metastasis in instances of THCA would facilitate the development of a more scientifically-informed treatment plan, enable regular assessment of patient prognosis, prompt timely treatment adjustments, and ultimately enhance patient prognosis.

In the case of THCA, several known risk factors have been linked to LNM, such as patient age, sex, multifocality, calcification, and extrathyroidal extension (ETE) (5, 10–12). In addition to established clinical factors, there has been a burgeoning interest in exploring genetic variations associated with LNM in recent years (13, 14). For example, experimental evidence from both *in vitro* and *in vivo* studies has demonstrated that the upregulation of lnc-MPEG1-1:1 in papillary thyroid cancer (PTC) cell lines can elevate cell proliferation and migration (15). Moreover, this long non-coding RNA (lncRNA) is observed to be overexpressed in the cytoplasm of PTC cells and has been shown to exert its function by acting as a competitive endogenous RNA (ceRNA), competitively sequestering the shared binding sequences of miR-766-5p (15). In addition, researchers have reported that primary patients with positive lymph node status tend to exhibit relatively advanced TI-RADS levels and higher prevalence of the RET genetic alteration (16). Therefore, a comprehensive understanding and analysis of genomic alterations in THCA with LNM are necessary to advance the current knowledge of the underlying pathophysiology involved in the development and predisposition to LNM. Such enhanced understanding could potentially pave the way for the development of improved resources and novel strategies for the prevention and treatment of LNM (17, 18).

Endocrine-disrupting chemicals (EDCs) are exogenous compounds found in the environment that can emulate or impair the functioning of endogenous hormones (19, 20). EDCs have the ability to interfere with reproductive, neuroendocrine, cardiovascular, and metabolic function, resulting in compromised health outcomes (20). The extensive impact of EDCs on the progression and metastasis of tumors of endocrine organs has been widely documented. According to a recent study report, bisphenol A (BPA), a kind of EDCs, has a promotional effect on breast ductal carcinoma *in situ* (DCIS) cell proliferation and migration, as well as macrophage migration (21). When exposed to an orally-administered, environmentally human-relevant low dose of 2.5 μg/l BPA for 70 days through drinking water in a DCIS xenograft model, primary tumor growth rate was promoted approximately 2-fold and lymph node metastasis was significantly increased, along with a notable enhancement of CD206+ M2 macrophage polarization, indicating a protumorigenic response. These findings reveal the role of BPA as an accelerator in advancing DCIS progression into invasive breast cancer by influencing DCIS cellular activity and macrophage polarization toward a cancer-supporting phenotype (21). Moreover, Tamoxifen, being an EDC, is widely used as a hormone therapy in postmenopausal women with breast cancer who are ER+ and is regarded as one of the most effective adjuvant breast cancer treatments available (22). Its effectiveness in controlling breast cancer recurrence and metastasis has been extensively reported. Previous studies have revealed the potential role of EDCs in THCA. Existing literature has revealed that exposure to certain congeners of flame retardants, polychlorinated biphenyls (PCBs), phthalates, and specific isomers of pesticides can lead to an increased risk of thyroid cancer (23). Exposure to Bisphenol A (BPA) has been associated with an increased risk of thyroid nodules in Chinese women (24). Additionally, animal experiments have demonstrated a correlation between BPA exposure and the risk of thyroid cancer (25). Despite THCA being the most frequent type of endocrine tumor, there has not been widespread research into the impact of EDCs on the LNM of THCA. Therefore, utilizing bioinformatics to investigate EDCs relevant to LNM in THCA is advantageous for further screening of potential therapeutic drugs and improving patient prognosis.

In light of the recent progress in high-throughput sequencing technology, the integration of multiple omics analysis has gained widespread utilization in tumor research (26–28). The high-throughput sequencing technology is capable of exploring tumor biomarkers, evaluating therapeutic responsiveness, and providing convenience for the development of clinical management plans among tumor patients (29–32). Therefore, the aim of this study is to comprehensively investigate the key genetic variations and EDCs relevant to LNM in THCA using multiple bioinformatics techniques. Additionally, we aim to screen for potential therapeutic drugs and corresponding treatment targets capable of inhibiting the incidence of LNM in THCA.

## Materials and methods

### Data acquisition

The clinical data, RNA-seq data, 450K methylation data, and copy number variation (CNV) data pertaining to the THCA (THCA) cohort were extracted from the GDC database (https://portal.gdc.cancer.gov/projects/TCGA-THCA) (33). A total of 510 THCA specimens, along with 58 normal specimens, were identified in the TCGA-THCA cohort. After obtaining the RNA-seq FPKM dataset, we proceeded to transform the expression profile into transcripts per kilobase million (TPM). The GSE60542 cohort, comprising 33 primary thyroid tumor samples, 23 metastatic lymph nodes, 30 normal thyroid samples, and 4 normal lymph node samples, was extracted from the Gene Expression Omnibus (GEO) database (http://www.ncbi.nlm.nih.gov/geo/), and it served as the validation cohort (34).

Gene Expression Profiling Interactive Analysis (GEPIA) database was used to obtain the differentially expressed genes (DEGs) between THCA and normal tissues (35). The criterion for screening DEGs is that the |Log_2_FC|>1 and *q*-value<0.05. The DEGs were also plotted as chromosomal distribution via GEPIA database.

### Identification of the potential for tumors to undergo lymph node metastasis

Our study introduces a novel concept called ‘LNM potential. ‘ In cases where a thyroid cancer patient experiences LNM with a small primary tumor volume, they are considered to have a high LNM potential. Conversely, if a thyroid cancer patient does not experience LNM despite having a larger primary tumor volume, they are considered to have a low LNM potential. In the TCGA-THCA cohort, patients with a tumor size exceeding the median but without LNM were classified as having low LNM potential (LNM Low), while patients with a tumor size below the median but with LNM were classified as having high LNM potential (LNM High).

### Weighted correlation network analysis

The transcriptional profiles of the DEGs obtained from GEPIA database were used as the input file for the R package “WGCNA” to establish the co-expression networks (36). WGCNA was performed with the default-recommended parameters. To distinguish modules with different expression patterns, a soft threshold power obtained from “pickSoftThreshold” algorithm was used for creating co-expression networks. The minimum module size was set to 30, and the dissimilarity threshold for module merging was set to 0.25. Pearsons correlation analysis were carried out to estimate correlation between Module eigengenes (MEs) and clinical traits and then the module with the highest and lowest pearsons coefficient was identified as the module most relevant to clinical traits.

### Identification of the hub genes

The online database STRING was employed to formulate the Protein-Protein Interaction (PPI) Network for all the genes in the module most relevant to clinical traits (37). Default setting was used in STRING database. The visual representation of the PPI network was accomplished through the Cytoscape tool (Version 3.7.2). The CytoNCA plugin, integrated with the Cytoscape tool, was utilized for determining the topological features and degree centralities of each node (protein) within the PPI network (38). Subsequently, the hub genes was singled out and delineated as the prominent node of the PPI network, crucial for mediating protein-protein interactions.

The hub gene-miRNA, Transcription factor (TF)-hub gene and TF-miRNA interactions was established using NetworkAnalyst online tool based on ENCODE database (http://www.encodeproject.org/ENCODE/), miRTarBase (v8.0; http://mirtarbase.mbc.nctu.edu/) and Regulatory Network Repository (https://regnetworkweb.org/) (39–42).

### Pathway enrichment analysis and immune infiltration analysis

Conducting pathway and process enrichment analyses was accomplished through employment of the Metascape platform (43) (Metascape, http://metascape.org). By following the default settings, the Metascape tool facilitated hierarchical clustering to segregate enrichment terms into unique clusters, with the representative term being selected based on minimal p-value criteria.

In order to ascertain the relative enrichment of a gene set in the given sample population, gene set variance analysis (GSVA) was implemented (44). The higher scores indicate a relatively greater activation of the gene set in the given sample. In this study, 10 cancer-associated pathways’ activity scores were computed for 7876 samples collected from 32 cancer types using the Reverse Phase Protein Array (RPPA) data derived from the TCPA database and the TCGA database (45). The pathways examined in this study are TSC/mTOR, RTK (receptor tyrosine kinase), RAS/MAPK, PI3K/AKT, Hormone ER, Hormone AR, EMT (epithelial-mesenchymal transition), DNA Damage Response, Cell Cycle, and Apoptosis pathways, all of which are well-known pathways associated with cancer. RPPA is a high-throughput antibody-based technology that involves procedures analogous to those of Western blots (46). In this technique, the proteins are extracted from cancerous tissue or cultured cells, denatured with SDS, and then immobilized on nitrocellulose-coated slides. Next, an antibody probe is used for analysis. Utilizing the Gene Set Cancer Analysis (GSCA) tool, the aforementioned analytical process was carried out to compute a pathway activity score (PAS) that effectively represents activation levels of the respective signaling pathway (47).

Immune Cell Abundance Identifier (ImmuCellAI) database was utilized to evaluate immune cell infiltration in each sample of TCGA-THCA cohort (48). The aforementioned tool was developed to assess the abundance of 24 immune cells within a given gene expression dataset, including RNA-Seq and microarray data. The 24 immune cells encompass 18 T-cell subtypes, as well as an additional six immune cells, specifically, B cells, NK cells, monocytes, macrophages, neutrophils, and DC cells.

### Recognition of molecular subtypes

Unsupervised hierarchical clustering of the hub genes was established by R package “ConsensusClusterPlus” to identify the different molecular subtypes in TCGA-THCA cohort (49). ConsensusClusterPlus was executed with default settings for all parameters, with the maximum evaluated ‘k’ (max K) restricted to 10. The optimal number of clusters (‘k’) was determined using the Consensus Cumulative Distribution Function (CDF) Plot. Visualization of the expression patterns of hub genes across different molecular subtypes was performed using the R package ‘pheatmap,’ with a heatmap-type display.

### Machine learning framework

In the TCGA-THCA cohort, a comprehensive analysis was conducted to identify key gene from the hub genes of PPI network utilizing the Least Absolute Shrinkage and Selection Operator (LASSO), Support Vector Machine (SVM), and the Random Forest (RF) algorithms available in the “glmnet”, “e1071”, and “randomForest” R packages, respectively (50–55). The application of these machine learning techniques enabled the effective screening of genes with potential diagnostic significance in the context of the studied cohort.

In order to perform LASSO algorithmic analysis, a set of specific parameters were established, including the family parameter, set to “binomial”, alpha parameter which was set to 1, type measure parameter defined as “deviance”, as well as the nfolds parameter set to 10 (31). For the construction of a forest of 500 trees, the “randomForest” package within R was effectively utilized through standard settings (29). Additionally, feature importance scores were calculated through the application of the “importance” function, which was performed through the utilization of the “randomForest” package in R. Following the implementation of randomForest algorithms, genes exhibiting an importance value exceeding the median were selected and subjected to downstream analysis. The SVM method ran using the default parameters. Through cross-referencing the results generated by the three methodologies, an intersectional subset was identified as the key gene set (30).

### Comparative toxicogenomics database

The publicly accessible CTD database (http://ctdbase.org/) is a comprehensive repository of toxicogenomic data, offering reliable and meticulously scrutinized information regarding gene/protein interactions with chemicals across an extensive range of peer-reviewed scientific literature (56). This trustworthy and vigorous database serves as a valuable platform for researchers seeking to access critical toxicogenomic information. Against the backdrop of default parameters, the CTD database is utilized to explore the potency of EDCs, antineoplastic drugs, and environmental toxins in their ability to incite changes in key gene expression within all species. Dependable EDCs were sourced from previously published literature (19).

### Discovery of potential drugs by computational methods

Drug sensitivity of anticancer drugs was estimated in each tumor specimen of TCGA-THCA by R package “oncoPredict” (57). Ridge regression was performed by “oncoPredict” algorithm and all above prediction was performed based on the Genomics of Drug Sensitivity in Cancer (GDSC) database (58).

### Molecular docking procedure

To obtain the crystal structures of proteins encoded by the hub gene, the RCSB Protein Data Bank (PDB) (www.rcsb.org/pdb/home/home.do) was accessed, while the 3D structures of the drugs were downloaded from PubChem (https://www.ncbi.nlm.nih.gov/pccompound) (59, 60). The molecular docking process was conducted using mcule 1-click Docking server online (https://mcule.com/apps/1-click-docking/) (61). The best pose was selected based on the docking score and the rationality of the molecular conformation.

### Exploration of protein expression level and subcellular localization of the key gene

The Human Protein Atlas (HPA) database (https://www.proteinatlas.org/), a comprehensive collection of human proteins in normal and tumor cells and tissues, integrates multiple cutting-edge omics technologies, including immunohistochemistry (IHC) and immunofluorescence (IF) (62). We employed the HPA online tool to investigate protein expression profiles of specific genes in both normal and tumor tissues, utilizing the immunohistochemistry data available in the HPA database.

Using the subcellular domain of the HPA database, we gained a high-resolution understanding of the spatiotemporal distribution and expression of proteins. Subcellular protein localization was investigated through immunofluorescence (ICC-IF) and confocal microscopy, involving up to three distinct cell lines. Based on image analysis, protein subcellular localization was systematically categorized into distinct organelles and intricately detailed subcellular structures.

### Real time quantitative PCR and IHC

Total RNA extraction was performed utilizing TRIzol reagent (Ambion, USA), followed by conversion of the extracted mRNA to cDNA using PrimeScript™ RT Master Mix (Takara, Japan). The gene transcripts were quantified through RT-qPCR assay utilizing ChamQ SYBR qPCR Master Mix (Vazyme, China). The 2-ΔΔCT method was used to evaluate the relative expression levels of the genes, with GAPDH serving as the internal reference. To detect ERBB3 and GAPDH expression levels, the forward primer of ERBB3 was 5′-GCAGATCAGTGTGTAGCGTG-3′, and the reverse primer of ERBB3 was 5′-CGTGTGCAGTTGAAGTGACA-3′; while the forward primer of GAPDH was 5′-TGTTCGTCATGGGTGTGAAC-3′ and the reverse primer of GAPDH was 5′-ATGGCATGGACTGTGGTCAT-3′. The experiment was repeated thrice for establishing the average. Gene expression was detected utilizing the RT-qPCR method.

The tumors were fixed in 4% paraformaldehyde and embedded in paraffin. Subsequently, 4 μm sections were obtained from the paraffin-embedded samples and fixed on glass slides. Epitope retrieval of the sections was performed in 10 mmol/L citric acid buffer at pH7.2, heated in a microwave. Following epitope retrieval, the slides were incubated at 4°C overnight with the primary antibody (rabbit anti-ERBB3, dilution 1:100, K113334P, Solarbio; Beijing, CN), followed by HRP-conjugated secondary antibody for 1 h at room temperature. The detection of antibodies was done using the substrate diaminobenzidine (DAB, Beyotime), and slides were counterstained with hematoxylin (Beyotime). For statistical analysis, Average Optical Density (AOD) was used as a scoring method. AOD measurements were executed by professional pathologists using the ImageJ software, and at least three measurements were taken per IHC sample to establish the mean AOD values.

The study utilized samples from 9 THCA patients without LNM and 11 patients with LNM from The Third Affiliated Hospital of Anhui Medical University. The samples were employed for RT-qPCR and IHC analyses. All patients involved in the study provided informed consent prior to their inclusion in the study.

### Statistical analyses

For statistical analysis, we employed R software (version 4.2.1). To compare continuous variables, the Wilcoxon/Kruskal-Wallis Test was utilized, whereas differences in proportion were assessed by the Chi-Square test. A p-value of less than 0.05 was regarded as statistically significant. For evaluation of diagnostic performance, the Receiver Operating Characteristic (ROC) curve was employed. Correlations were analyzed using Spearman’s correlation. T-Distribution Stochastic Neighbor Embedding (t-SNE), uniform manifold approximation and projection (UMAP), and principal component analysis (PCA) were employed for dimensionality reduction (63–65).

## Results

### Alterations in biological processes and immune cell infiltration associated with LNM in thyroid cancer

The median tumor diameter in the TCGA-THCA cohort was 2.5cm. There were 99 cases of patients (LMN Low) with tumor diameter exceeding 2.5cm but no LNM, and 88 cases of patients (LMN High) with tumor diameter below 2.5cm but with LNM. The **Table 1** presented patient clinical characteristics.

**Table 1:** The clinical data from enrolled patients into the study.

Characteristics	LNM High(N=88)	LNM Low(N=99)	Total(N=187)	pvalue	FDR
Age				0.1	0.58
<=46	39(20.86%)	57(30.48%)	96(51.34%)		
>46	49(26.20%)	42(22.46%)	91(48.66%)		
Sex				1	1
FEMALE	63(33.69%)	71(37.97%)	134(71.66%)		
MALE	25(13.37%)	28(14.97%)	53(28.34%)		
Primary neoplasm				3.20E-03	0.02
Multifocal	52(27.81%)	34(18.18%)	86(45.99%)		
Unifocal	35(18.72%)	63(33.69%)	98(52.41%)		
unknow	1(0.53%)	2(1.07%)	3(1.60%)		
T				3.00E-09	2.70E-08
T1	40(21.39%)	7(3.74%)	47(25.13%)		
T2	17(9.09%)	53(28.34%)	70(37.43%)		
T3	27(14.44%)	36(19.25%)	63(33.69%)		
T4	4(2.14%)	3(1.60%)	7(3.74%)		
N				1.10E-41	1.10E-40
N0	0(0.0e+0%)	99(52.94%)	99(52.94%)		
N1	88(47.06%)	0(0.0e+0%)	88(47.06%)		
M				0.67	1
M0	51(27.27%)	57(30.48%)	108(57.75%)		
M1	1(0.53%)	3(1.60%)	4(2.14%)		
unknow	36(19.25%)	39(20.86%)	75(40.11%)		
Stage				2.00E-07	1.60E-06
Stage I	45(24.06%)	43(22.99%)	88(47.06%)		
Stage II	1(0.53%)	30(16.04%)	31(16.58%)		
Stage III	24(12.83%)	21(11.23%)	45(24.06%)		
Stage IV	18(9.63%)	4(2.14%)	22(11.76%)		
unknow	0(0.0e+0%)	1(0.53%)	1(0.53%)		
Location				0.28	1
Bilateral	19(10.16%)	14(7.49%)	33(17.65%)		
Isthmus	6(3.21%)	4(2.14%)	10(5.35%)		
Left lobe	27(14.44%)	25(13.37%)	52(27.81%)		
Right lobe	35(18.72%)	55(29.41%)	90(48.13%)		
unknow	1(0.53%)	1(0.53%)	2(1.07%)		
Residual tumor				0.17	0.87
R0	65(34.76%)	79(42.25%)	144(77.01%)		
R1	12(6.42%)	5(2.67%)	17(9.09%)		
R2	0(0.0e+0%)	1(0.53%)	1(0.53%)		
unknow	11(5.88%)	14(7.49%)	25(13.37%)		
Thyroid gland disorder history				0.43	1
No	50(26.74%)	47(25.13%)	97(51.87%)		
Yes	27(14.44%)	38(20.32%)	65(34.76%)		
unknow	11(5.88%)	14(7.49%)	25(13.37%)		

Differential gene analysis of the LMN High and LMN Low groups was performed using the limma R package, with a screening criterion of |Log2FC| > 1 and *p*-value < 0.05. A total of 1038 upregulated genes and 332 downregulated genes were identified in the LMN High group of patients (**Supplementary Table 1**). Pathway enrichment analysis was performed on upregulated and downregulated genes separately, revealing that the upregulated genes were mainly enriched in adaptive immune response, NABA MATRISOME ASSOCIATED, and positive regulation of immune response. Meanwhile, the downregulated genes were mainly enriched in positive regulation of CoA-transferase activity, Metallothioneins bind metals, and monoatomic ion transmembrane transport (**Supplementary Figures 1A, B**).

Moreover, there were significant differences in immune infiltration status between the LMN High and LMN Low groups (**Supplementary Table 2**). Specifically, nTreg, iTreg, Th1, and CD8T cells exhibited relatively higher infiltration levels in the LMN High group, while neutrophils exhibited relatively higher infiltration levels in the LMN Low group (**Supplementary Figure 1C**). Out of the 10 cancer-related pathways obtained using RPPA technology, only the PI3K/AKT, TSC/mTOR, and RTK pathways were found to have significantly lower activation levels in the LMN High group compared to the LMN Low group (**Supplementary Figure 1D**).

### LNM potential-related gene module revealed by WGCNA

**Figure 1:**
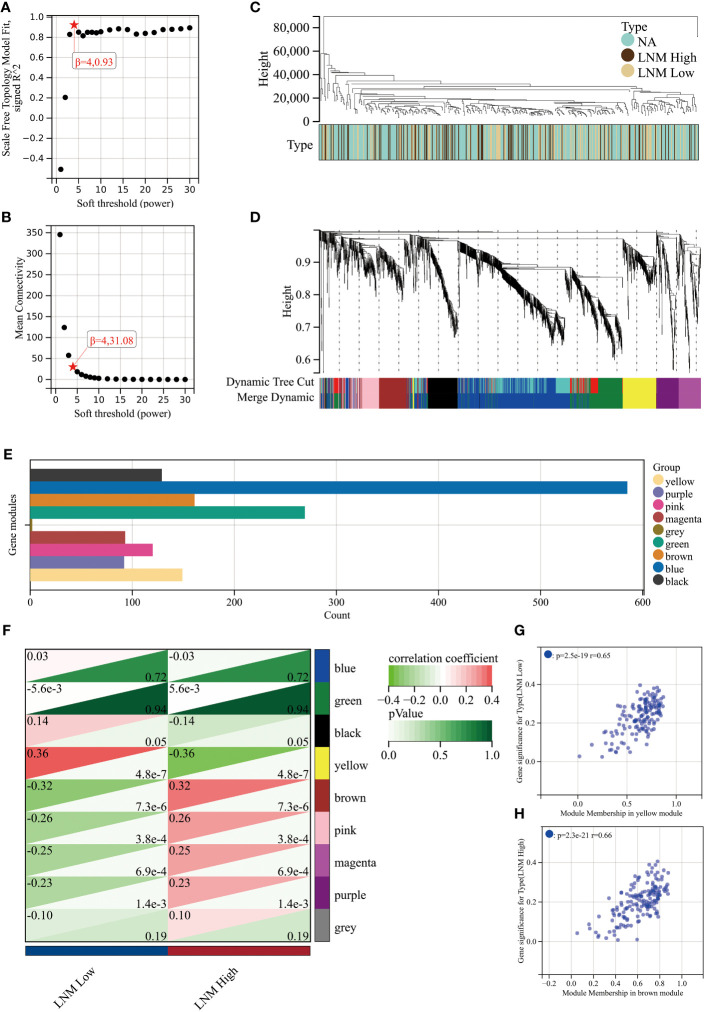
An investigation into the determination of soft-thresholding power used in WGCNA. **(A)** An examination of the scale-free fit index for different soft-thresholding powers (β). **(B)** Investigation into the mean connectivity for different soft-thresholding powers. **(C)** Illustration of the sample dendrogram and clustering dendrogram via WGCNA. **(D)** Hierarchical cluster tree depicting the co-expression modules discovered through WGCNA. **(E)** The number of genes in different gene modules. **(F)** The correlation between different gene modules and the LNM potential. The correlation between module membership (MM) and gene significance (GS) in the yellow **(G)** and brown **(H)** modules.

**Figure 2:**
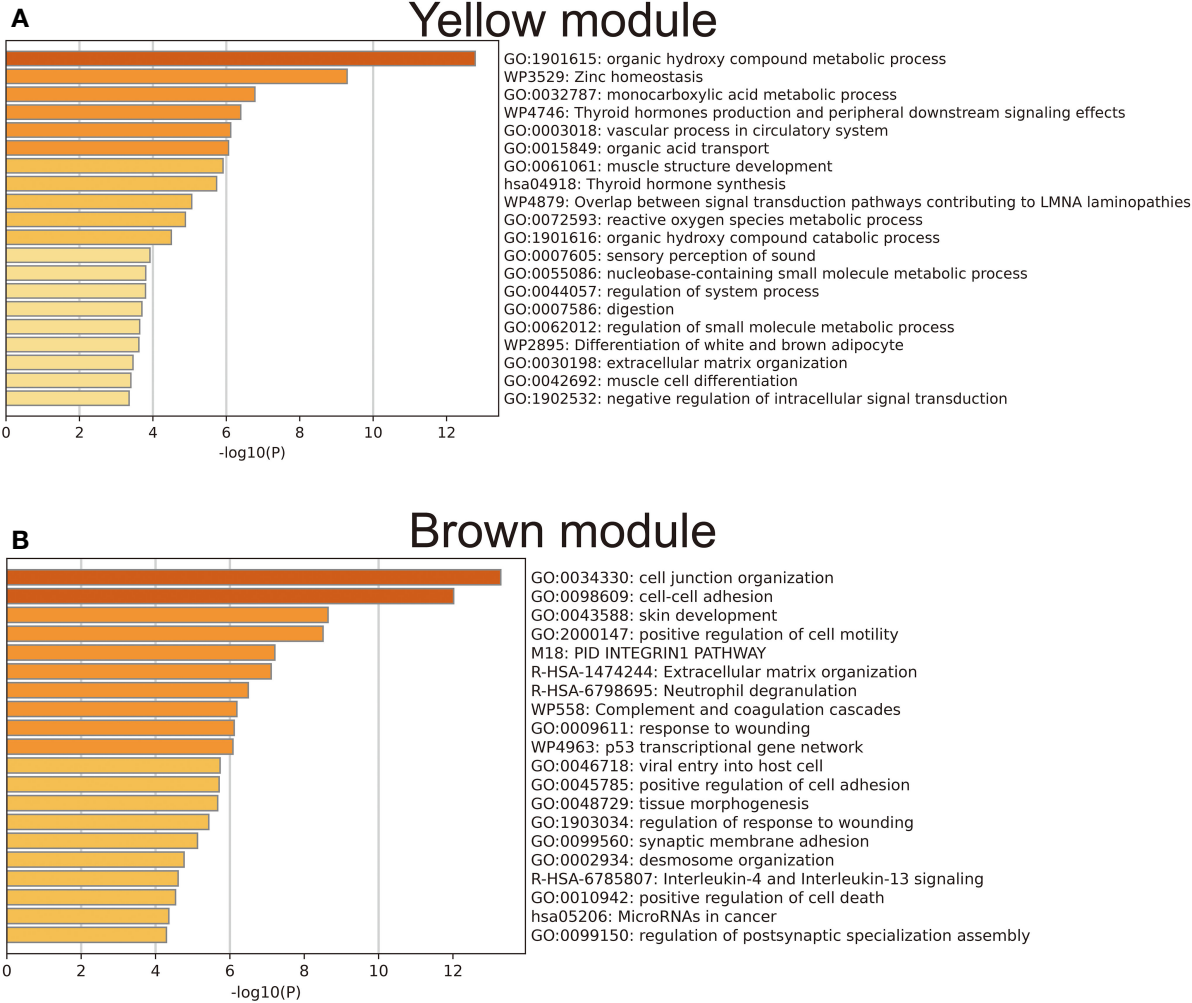
Results of gene enrichment analysis for the yellow **(A)** and brown **(B)** gene modules.

To achieve a signed scale-free co-expression gene network, a power of β=4 and a scale-free R2 = 0.93 were chosen as the soft-threshold parameters (**Figures 1A, B**). Within the context of WGCNA analysis, sample clustering was conducted utilizing gene expression patterns in order to identify outliers (**Figure 1C**). Consequently, 9 gene modules were successfully delineated in the TCGA-THCA cohort (**Figure 1D**; **Supplementary Table 3**). The “grey” module was created to encompass genes that could not be sorted into any other discernible genetic module. The module with the greatest number of included genes was the “blue” module (n=585), while the “grey” module (n=2) contained the fewest number of included genes (**Figure 1E**). The calculation of correlation between module eigengenes (MEs) and clinical features was conducted using the Pearson’s correlation analysis. Through this analytical process, it was discovered that the “brown” module displayed the highest positive correlation with LMN High, while conversely, the “yellow” module showed the highest negative correlation with LMN High (**Figure 1F**). The significant correlation observed between GS and MM within both the “brown” and “yellow” modules suggests a strong association between these modules and the potential for LNM (**Figures 1G, H**). The biological processes primarily enriched by genes within the “yellow” module included organic hydroxy compound metabolic process, homeostasis, and monocarboxylic acid metabolic process, among others (**Figure 2A**). The “brown” module was primarily enriched in genes associated with biological processes such as cell junction organization, cell-cell adhesion, skin development, and positive regulation of cell motility (**Figure 2B**).

### Identification of hub genes in the LNM potential-related gene modules

**Figure 3:**
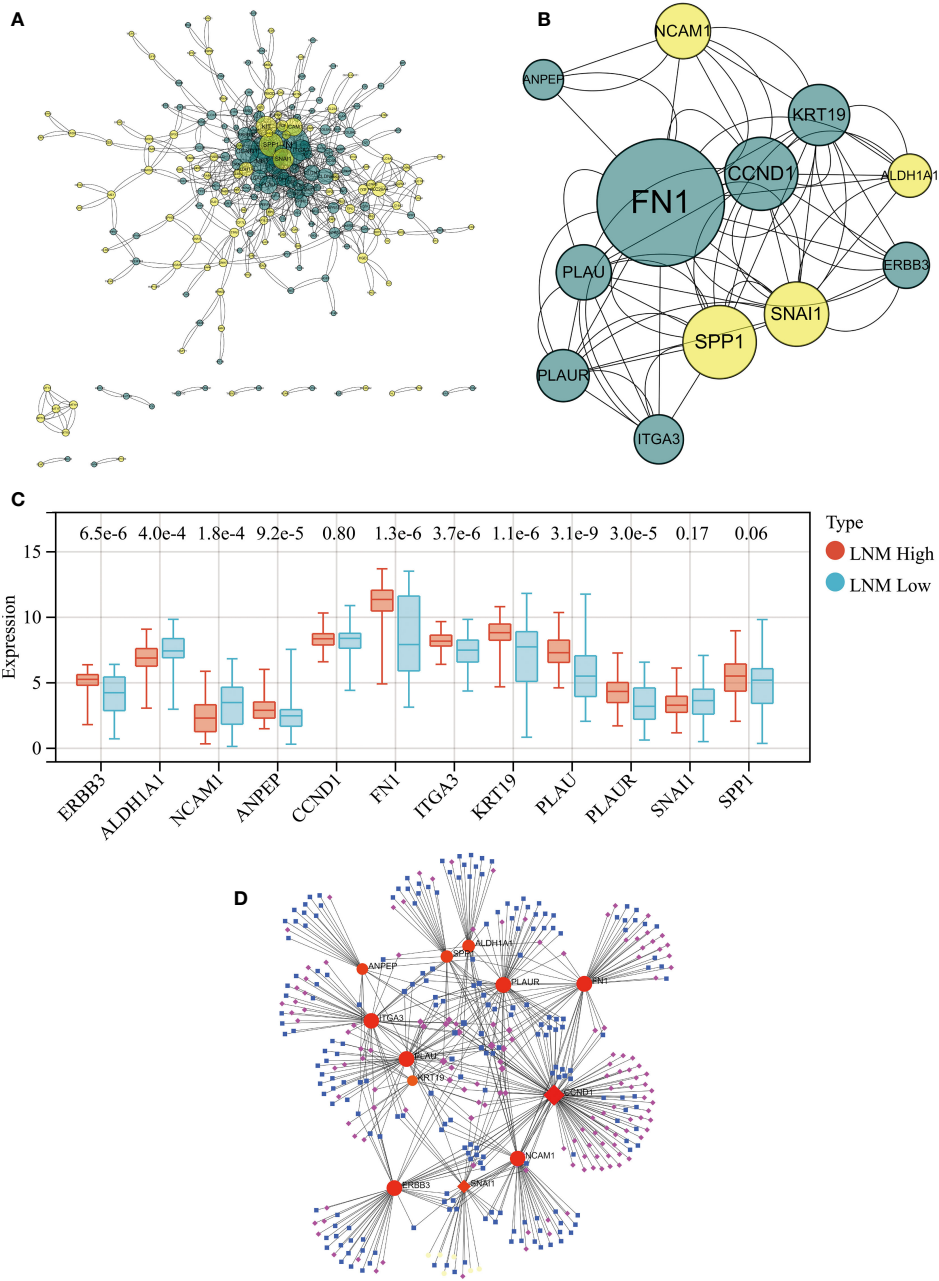
**(A)** PPI network for all genes within the yellow and brown gene modules. **(B)** The PPI network’s hub genes were screened through the use of CytoNCA, with the top 12 hub genes being further selected via CytoNCA. **(C)** Expression levels of these 12 hub genes in THCA patients with high and low LNM potential. **(D)** Gene-miRNA, TF-gene, and TF-miRNA interaction networks centered around these 12 hub genes.

PPI network analysis of all genes within the ‘yellow’ and ‘brown’ modules was performed using the STRING tool (**Figure 3A**). A key cluster (Cluster 1) of the PPI network was extracted using the CytoNCA plugin within the Cytoscape software, and ERBB3 served as the seed of this cluster (**Supplementary Table 4**). The identified cluster consisted of 12 hub genes related to LNM potential, with 4 of them originating from the ‘yellow’ gene module and the rest from the ‘brown’ module (**Figure 3B**).

Expression levels of ALDH1A1 and NCAM1 were observed to be upregulated in the LNM Low group, whereas PLAU, KRT19, FN1, ITGA3, ERBB3, PLAUR, and ANPEP were found to be overexpressed in the LNM High group (**Figure 3C**; **Supplementary Table 5**). Using the 12 hub genes as the central framework, we constructed gene-miRNA, TF-gene, and TF-miRNA interaction networks to investigate the key regulatory mechanisms underlying gene expression (**Figure 3D**; **Supplementary Table 6**).

### The diagnostic ability of hub genes in THCA

**Figure 4:**
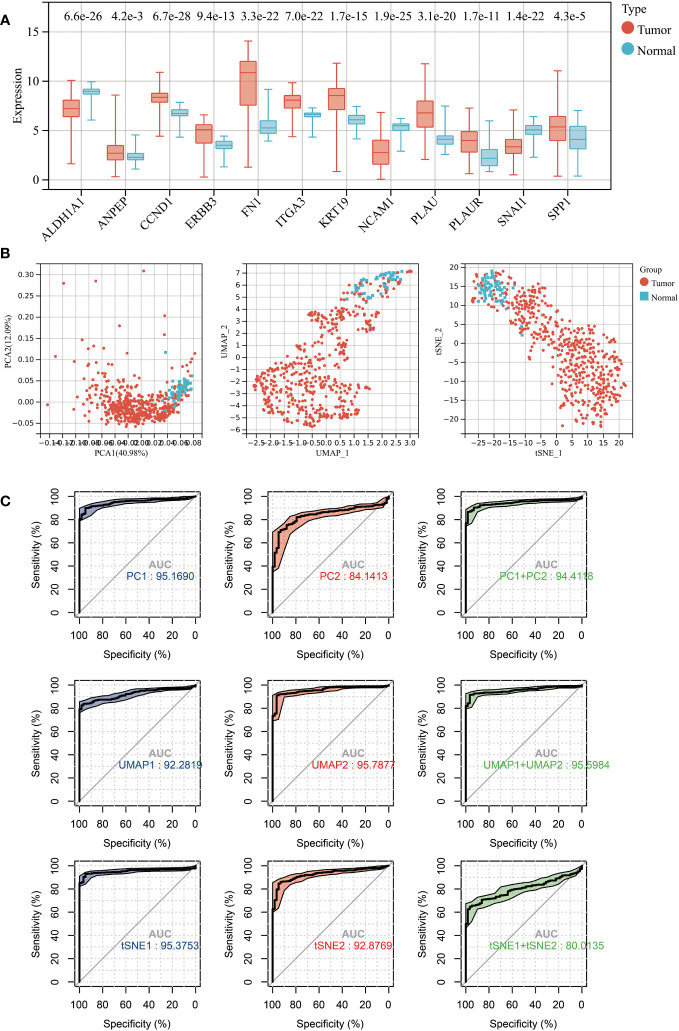
**(A)** Expression levels of 12 LNM potential-related hub genes between THCA and normal thyroid tissue. **(B)** The PCA (left), UMAP (middle), and TSNE (right) dimensionality reduction algorithms were utilized to generate data visualization. **(C)** The diagnostic capability of various dimensionality reduction algorithms on THCA was evaluated via ROC plot, utilizing the first two principal components and the sum of the first and second principal components.

All 12 hub genes related to LNM potential exhibited significant differential expression between THCA and normal thyroid tissues (**Figure 4A**). Specifically, the gene expressions of ALDH1A1, NCAM1, and SNAI1 were downregulated in THCA, while the expressions of the remaining nine genes were upregulated.

Subsequently, we conducted dimensional reduction analysis based on hub gene expression using PCA, UMAP, and t-SNE. These analyses effectively distinguished THCA from normal tissues (**Figure 4B**). ROC analysis demonstrated that PCA1/2, UMAP1/2, t-SNE1/2, and their combination can serve as outstanding diagnostic biomarkers for THCA (**Figures 4B, C**).

### The variations in immune infiltration and pathway activation associated to LNM potential-related hub genes

**Figure 5:**
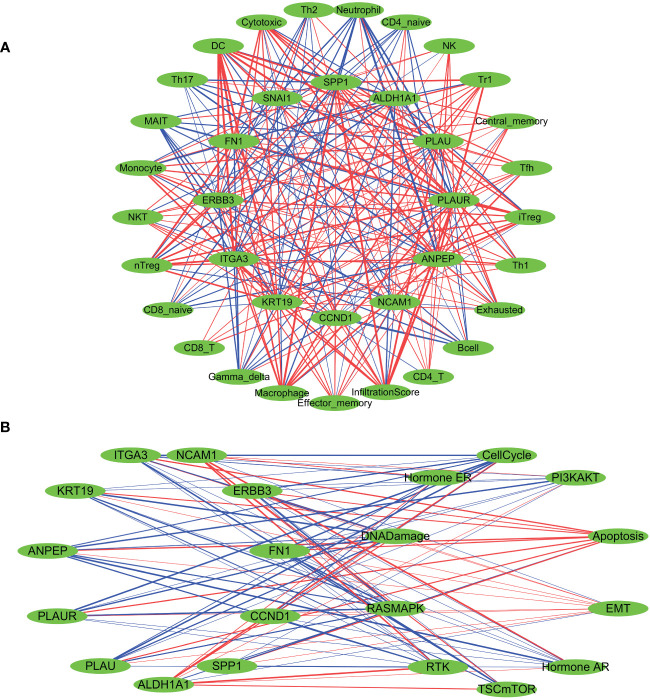
Spearman’s correlation analysis was performed to evaluate the correlation between the expression levels of LNM potential-related hub genes and tumor immune cell infiltration **(A)**, as well as the ten cancer-related pathways **(B)**. The red lines indicate positive correlation, the blue lines indicate negative correlation, and the thickness of the lines represents the correlation coefficient.

A Spearman correlation analysis was performed to investigate the correlation between the gene expression levels of all 12 LNM potential-related hub genes and the infiltration scores of different immune cells (**Figure 5A**; **Supplementary Table 7**). With the exceptions of SNAI1, NCAM1, and ALDH1A1, the infiltration levels of DC cells showed significant positive correlations with other hub genes, with R>0.5 and *p*<0.0001. Of particular note was the strongest positive correlation observed between the infiltration levels of DC cells and the gene expression levels of FN1 (R=0.77; *p*<0.001). Furthermore, there was a significant negative correlation between the gene expression levels of DC cells and ALDH1A1 (R=-0.58; *p*<0.0001). Neutrophil infiltration levels did not show a significant correlation with SNAI1 and CCND1. The correlation observed between Neutrophil infiltration levels and NCAM1 was weakly positive (R=0.17; *p <*0.0001). In addition, there were significant negative correlations observed between Neutrophil infiltration levels and the other nine identified hub genes, with ANPEP exhibiting the strongest negative correlation (R=-0.69; *p <*0.0001).

Subsequently, we investigated the influence of CNV and SNV status of hub genes on immune cell infiltration in tumors. A sample was classified as either CNV-Amplification (Amp), CNV-Deletion (Del), or SNV-Mutant based on the occurrence of a CNV or SNV alteration in at least one of the identified hub genes. Using a significance level of P<0.05 as a filtering criterion, it was observed that the occurrence of CNV Amplification in hub genes was associated with a relatively higher degree of variability in immune cell infiltration, compared to CNV Deletion and SNV-Mutant (**Supplementary Figures 3A, B**). Furthermore, we conducted an evaluation of the influence of the activation levels of identified hub genes (GSVA scores) on immune cell infiltration in various cancer types using pan-cancer analysis based on the GSVA algorithm. This analysis encompassed assessments across 33 cancer types (**Supplementary Figure 3C**; **Supplementary Table 8**). A positive correlation was observed between the activation levels of identified hub genes and the levels of DC and macrophage infiltration in the majority of the analyzed tumor types. In contrast, a negative correlation was noted between hub gene activation levels and the level of neutrophil infiltration.Similar results were observed in the TCGA-THCA cohort, where a strong positive correlation was found between the GSVA scores of identified hub genes and the level of DC infiltration. Simultaneously, a robust negative correlation was identified between hub gene GSVA scores and the level of neutrophil infiltration (**Supplementary Figure 3D**).

Based on the median gene expression of hub genes, the samples were segregated into two groups – High and Low. To determine the difference in PAS score between the groups, the student T test was performed and the *p*-value was adjusted by false discovery rate (FDR). We considered a gene to have an activating effect on a pathway if the FDR PAS (gene A Low expression) value suggested so (FDR<0.05), and conversely, we classified it as having an inhibitory effect. A similar methodology was employed by Y. Ye et al. (66). The results of the TCGA-THCA cohort highlighted a pronounced regulatory impact of hub genes on the EMT, PI3K-AKT, and RTK signaling pathways. The overexpression of NCAM1 and ALDH1A1 signifies a more inhibitory effect on the EMT pathway and an enhanced activation of the RTK and PI3K-AKT pathways. The activation of the EMT, RTK, and PI3K-AKT pathways are not significantly influenced by SNAI1, whereas CCND1 activates the EMT pathway while suppressing the RTK pathway. Elevated expression levels of the remaining hub genes indicate the activation of the EMT pathway and inhibition of the RTK and PI3K-AKT pathways (**Figure 5B**; **Supplementary Table 9**). Furthermore, a pancancer analysis was conducted to investigate the regulatory effects of different hub genes on cancer-associated pathways in various types of cancer, as demonstrated in **Supplementary Figure 3E**. The pancancer analysis revealed that these hub genes exhibit the highest advantage in activating the EMT pathway.

### The establishment of a molecular classification scheme

**Figure 6:**
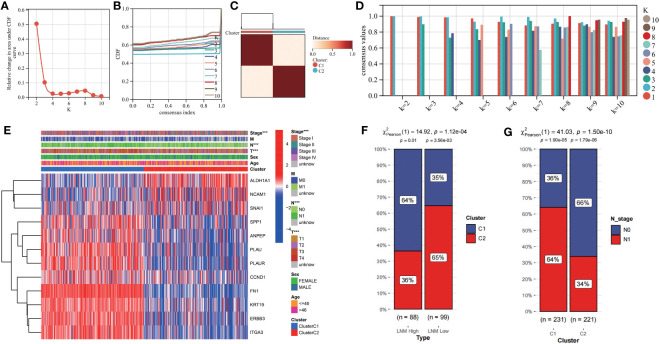
Constructing a novel molecular subtyping scheme using unsupervised clustering. **(A)** relative change in area under cumulative distribution function (CDF) curve. **(B)** Consensus clustering CDF for k=2-10. **(C)** Consensus matrix of THCA samples co-occurrence proportion for k = 2. **(D)** Cluster consensus values when K=1 to 10. **(E)** The expression levels of LNM potential-related hub genes between different clusters are shown in a heatmap. **(F)** The proportions ofpatients with high and low LNM potential in Cluster 1 and Cluster 2. **(G)** The proportions of patients with N0 and N1 staging in Cluster 1 and Cluster 2. ***: *p*<0.001.

To further integrate the features of the 12 identified hub genes for predicting LNM potential in THCA patients, we performed unsupervised clustering using “ConsensusClusterPlus”. Based on the consensus CDF and relative changes in the area beneath the CDF curve, it was determined that all patients could be effectively clustered into two distinct groups (cluster 1 and cluster 2; **Figures 6A–D**). The heatmap revealed distinct gene expression patterns across different patient clusters (**Figure 6E**). Subsequently, we conducted further investigations into the relationship between the molecular classification scheme and LNM in THCA patients. In the TCGA-THCA cohort, patients with low LNM potential were found to be predominantly composed of individuals within cluster 2 (65%; chi-square test, chi-square value= 14.92, *p*-value<0.001; **Figure 6F**), whereas those within cluster 1 demonstrated a higher incidence of LNM (64%; chi-square test, chi-square value= 41.03, *p*-value<0.0001, **Figure 6G**).

### Establishment of an online nomogram tool for improved clinical decision making

**Figure 7:**
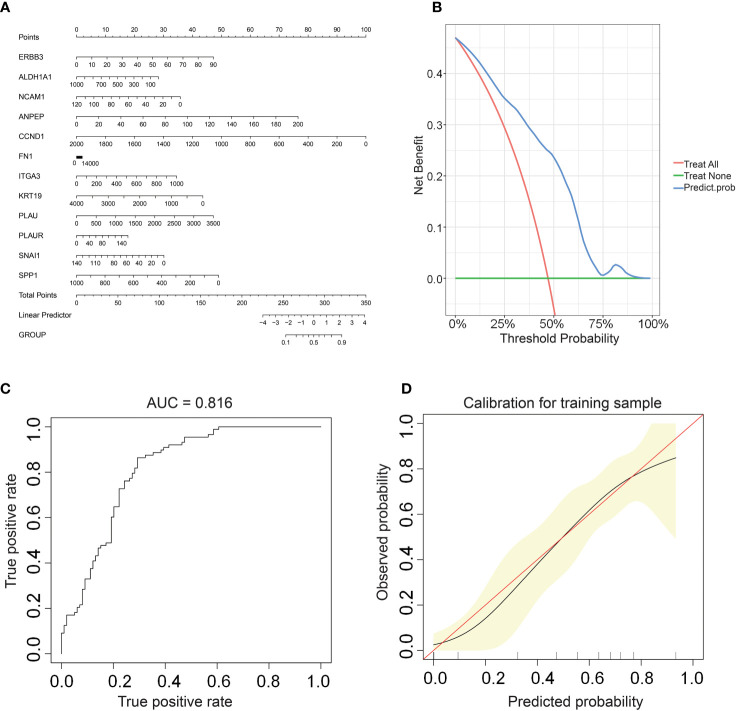
**(A)** A nomogram for predicting LNM potential in THCA. The DCA curve **(B)**, ROC curve **(C)**, and calibration curve **(D)** for the predictive nomogram.

We constructed a nomogram based on the gene expression levels of 12 hub genes that serves to assess the LNM potential of THCA patients (**Figure 7A**). Establishment of the nomogram was executed using the rms R package. Performance assessment of the nomogram was conducted using decision curve analysis (DCA) (**Figure 7B**), receiver operating characteristic curve (ROC) (**Figure 7C**), and calibration curve (**Figure 7D**). Clinical utility of the nomogram was confirmed by DCA. **Figure 7C** demonstrated that the area under the ROC curve (AUC) of the nomogram incorporating all predictors for high-LNM potential patients was 0.816. The calibration curve’s proximity to the ideal diagonal line was indicative of the good predictive performance of the nomogram. Furthermore, in order to further promote the accessibility and clinical utilization of our nomogram, it is noteworthy that an online web tool named “LNM potential” has been devised. The web address for this online tool is located at http://www.empowerstats.net/pmodel/?m=17617_LNM. By means of this online tool, the application of our research findings to the clinical setting may be further actualized. This tool contributes to the identification of THCA patients with a high LNM potential, providing a foundation for the development of individualized clinical treatment regimens.

### Further exploration based on machine learning to identify key genes associated with LNM potential

**Figure 8:**
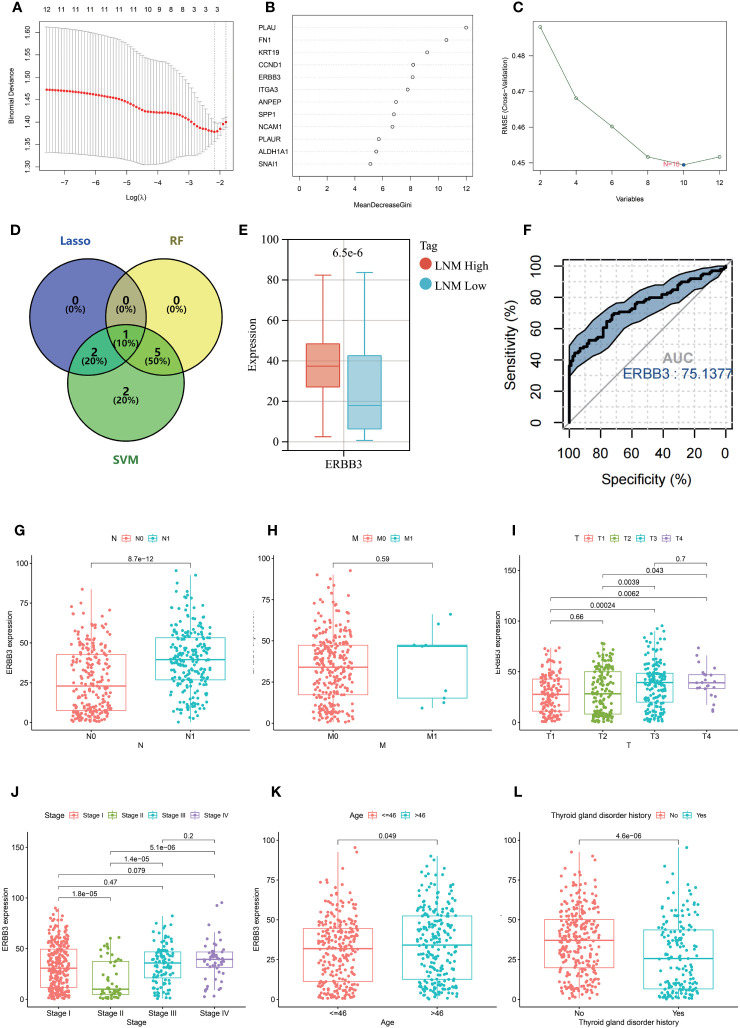
Results of selection by LASSO **(A)**, random forest **(B)**, and SVM **(C)**. **(D)** Venn diagram depicting the overlapping genes selected by LASSO, random forest, and SVM models. **(E)** The expression level of ERBB3 in individuals with different LNM potentials of THCA. **(F)** ROC analysis for the ability of ERBB3 to diagnose individuals with high LNM potentials of THCA. The expression level of ERBB3 has been examined in relation to different N stages **(G)**, M stages **(H)**, T stages **(I)**, tumor stages **(J)**, and ages **(K)**. **(L)** The gene expression levels of ERBB3 in patients with and without a history of Thyroid gland disorder.

Three machine learning methods (Lasso, Random forest, SVM) were employed to further screen key genes that could influence the LNM potential in patients with THCA from 12 hub genes (**Figures 8A–C**). ERBB3 was identified as being important for LNM potential in all three machine learning algorithms (**Figure 8D**). ERBB3 expression was upregulated in patients with high lymph node metastatic potential (LNM High) and ROC analysis indicated ERBB3 as a promising diagnostic biomarker for LNM High patients (**Figures 8E, F**).

### The relationship between ERBB3 mRNA expression and DNA methylation levels with different clinical features

Further investigation was conducted to explore the association between ERBB3 and various clinical characteristics (**Table 2**). ERBB3 was significantly upregulated in THCA patients with lymph node metastasis as well as those with higher T stage, but there was no significant difference in ERBB3 expression between M0 and M1 patients (**Figures 8G–I**). Patients in Stage II had the lowest level of ERBB3 expression (**Figure 8J**). It is noteworthy that patients of older age or with a medical history of thyroid gland disorder exhibited a significant upregulation of ERBB3 mRNA levels (**Figures 8K, L**). In the TCGA-THCA cohort, the variables of Sex, primary neoplasm location, and number did not significantly perturb the expression level of ERBB3 (**Supplementary Figures 4A–C**). ERBB3 has no impact on the complete surgical resection rate of THCA (**Supplementary Figure 4D**).

**Table 2:** Clinical information of patients with high and low ERBB3 mRNA expression levels.

Covariates	Type	Total	mRNA-High	mRNA-Low	Pvalue
Age	<=46	266(52.99%)	128(51%)	138(54.98%)	0.4209
	>46	236(47.01%)	123(49%)	113(45.02%)	
Sex	FEMALE	367(73.11%)	180(71.71%)	187(74.5%)	0.5459
	MALE	135(26.89%)	71(28.29%)	64(25.5%)	
Primary neoplasm	Multifocal	226(45.02%)	119(47.41%)	107(42.63%)	0.3618
	Unifocal	266(52.99%)	128(51%)	138(54.98%)	
	unknow	10(1.99%)	4(1.59%)	6(2.39%)	
T	T1	143(28.49%)	57(22.71%)	86(34.26%)	0
	T2	164(32.67%)	73(29.08%)	91(36.25%)	
	T3	170(33.86%)	103(41.04%)	67(26.69%)	
	T4	23(4.58%)	18(7.17%)	5(1.99%)	
	unknow	2(0.4%)	0(0%)	2(0.8%)	
N	N0	229(45.62%)	90(35.86%)	139(55.38%)	0
	N1	223(44.42%)	141(56.18%)	82(32.67%)	
	unknow	50(9.96%)	20(7.97%)	30(11.95%)	
M	M0	282(56.18%)	148(58.96%)	134(53.39%)	1
	M1	9(1.79%)	5(1.99%)	4(1.59%)	
	unknow	211(42.03%)	98(39.04%)	113(45.02%)	
Stage	Stage I	281(55.98%)	135(53.78%)	146(58.17%)	2.00E-04
	Stage II	52(10.36%)	16(6.37%)	36(14.34%)	
	Stage III	112(22.31%)	59(23.51%)	53(21.12%)	
	Stage IV	55(10.96%)	40(15.94%)	15(5.98%)	
	unknow	2(0.4%)	1(0.4%)	1(0.4%)	
Location	Bilateral	86(17.13%)	50(19.92%)	36(14.34%)	0.012
	Isthmus	22(4.38%)	14(5.58%)	8(3.19%)	
	Left lobe	175(34.86%)	95(37.85%)	80(31.87%)	
	Right lobe	213(42.43%)	89(35.46%)	124(49.4%)	
	unknow	6(1.2%)	3(1.2%)	3(1.2%)	
Residual tumor	R0	384(76.49%)	185(73.71%)	199(79.28%)	0.1177
	R1	52(10.36%)	32(12.75%)	20(7.97%)	
	R2	4(0.8%)	3(1.2%)	1(0.4%)	
	unknow	62(12.35%)	31(12.35%)	31(12.35%)	
Thyroid gland disorder history	Yes	165(32.87%)	69(27.49%)	96(38.25%)	0.0069
	No	279(55.58%)	155(61.75%)	124(49.4%)	
	unknow	58(11.55%)	27(10.76%)	31(12.35%)	

A pan-cancer analysis was conducted to investigate the DNA methylation levels of ERBB across various types of cancer (**Supplementary Figure 5A**). It was observed that the methylation levels of ERBB3 in the THCA samples were significantly lower than those in normal tissue, which partially explains the high expression of ERBB3 mRNA in THCA. The Shiny Methylation Analysis Resource Tool (SMART) was employed to annotate the methylation sites of ERBB3 (**Supplementary Figures 5B, C**). As anticipated, the methylation level of ERBB3 was notably higher in patients with low LNM potential, which could be a significant contributing factor impeding the expression level of ERBB3 mRNA in patients with low LNM potential (**Supplementary Figure 5D**; **Table 3**). Age and gender did not exhibit a significant effect on the degree of ERBB3 methylation (**Supplementary Figure 5E**). Patients who experienced LNM or were classified as T4 exhibited a diminished level of ERBB3 methylation, whereas stage II patients experienced an elevated amount of methylation. The occurrence of tumor metastasis, however, did not impact the degree of ERBB3 methylation (**Supplementary Figures 5G–J**). Furthermore, a tumor that develops in the isthmus or a patient with a history of thyroid gland disorder results in lower levels of ERBB3 methylation. The degree of ERBB3 methylation shows no significant correlation with the number of tumors or postoperative residual tumors (**Supplementary Figures 5K–N**).

**Table 3:** Clinical information of patients with high and low ERBB3 methylation levels.

Covariates	Type	Total	Methy-High	Methy-Low	Pvalue
Age	<=46	266(52.99%)	139(55.38%)	127(50.6%)	0.3253
	>46	236(47.01%)	112(44.62%)	124(49.4%)	
Sex	FEMALE	367(73.11%)	188(74.9%)	179(71.31%)	0.4207
	MALE	135(26.89%)	63(25.1%)	72(28.69%)	
Primary neoplasm	Multifocal	226(45.02%)	112(44.62%)	114(45.42%)	1
	Unifocal	266(52.99%)	132(52.59%)	134(53.39%)	
	unknow	10(1.99%)	7(2.79%)	3(1.2%)	
T	T1	143(28.49%)	80(31.87%)	63(25.1%)	0.0032
	T2	164(32.67%)	90(35.86%)	74(29.48%)	
	T3	170(33.86%)	74(29.48%)	96(38.25%)	
	T4	23(4.58%)	5(1.99%)	18(7.17%)	
	unknow	2(0.4%)	2(0.8%)	0(0%)	
N	N0	229(45.62%)	136(54.18%)	93(37.05%)	0
	N1	223(44.42%)	87(34.66%)	136(54.18%)	
	unknow	50(9.96%)	28(11.16%)	22(8.76%)	
M	M0	282(56.18%)	140(55.78%)	142(56.57%)	1
	M1	9(1.79%)	4(1.59%)	5(1.99%)	
	unknow	211(42.03%)	107(42.63%)	104(41.43%)	
Stage	Stage I	281(55.98%)	138(54.98%)	143(56.97%)	0.0011
	Stage II	52(10.36%)	37(14.74%)	15(5.98%)	
	Stage III	112(22.31%)	57(22.71%)	55(21.91%)	
	Stage IV	55(10.96%)	18(7.17%)	37(14.74%)	
	unknow	2(0.4%)	1(0.4%)	1(0.4%)	
Location	Bilateral	86(17.13%)	44(17.53%)	42(16.73%)	0.2743
	Isthmus	22(4.38%)	7(2.79%)	15(5.98%)	
	Left lobe	175(34.86%)	85(33.86%)	90(35.86%)	
	Right lobe	213(42.43%)	113(45.02%)	100(39.84%)	
	unknow	6(1.2%)	2(0.8%)	4(1.59%)	
Residual tumor	R0	384(76.49%)	185(73.71%)	199(79.28%)	0.5754
	R1	52(10.36%)	23(9.16%)	29(11.55%)	
	R2	4(0.8%)	1(0.4%)	3(1.2%)	
	unknow	62(12.35%)	42(16.73%)	20(7.97%)	
Thyroid gland disorder history	Yes	165(32.87%)	100(39.84%)	65(25.9%)	0
	No	279(55.58%)	110(43.82%)	169(67.33%)	
	unknow	58(11.55%)	41(16.33%)	17(6.77%)	

### Exploring EDCs, antineoplastic drugs, and environmental toxins that potentially influence the LNM potential

The thyroid gland is regarded as one of the most crucial endocrine organs. The endocrine system has been demonstrated to impact the metastasis and prognosis of various endocrine organ tumors. Hence, we aspire to investigate whether certain EDCs can impact the LNM potential of THCA. Our analysis of the CTD database revealed a potential interaction between 14 types of EDCs and the key gene ERBB3 that can affect ERBB3 mRNA expression, implying their indirect impact on the LNM potential of THCA. The 14 types of EDCs identified consist of Benzo(a)pyrene, bisphenol A, Estradiol, Genistein, Progesterone, Copper, Tamoxifen, Ethinyl Estradiol, Arsenic, Diethylstilbestrol, Androgen Antagonists, Cadmium, Raloxifene Hydrochloride, and Androgens (**Supplementary Table 10**).

Moreover, we have identified several antineoplastic drugs that are already in clinical use that can disturb the gene expression level of ERBB3. These drugs include Capecitabine, Doxorubicin, Epirubicin, Erlotinib Hydrochloride, Etoposide, Fluorouracil, Lapatinib, Mitomycin, and Paclitaxel (**Supplementary Table 10**). Therefore, we can speculate that these anticancer drugs may have the potential to reduce the LNM potential of THCA and could represent a potential therapeutic option for patients with thyroid cancer who have already undergone LNM. These findings will be further validated in the next chapter of this study.

Additionally, there are other drugs and environmental toxins that have been found to interact with ERBB3. Therefore, our study suggests that it would be beneficial for patients with THCA to avoid exposure to these toxins or use these drugs with caution, thereby contributing to the refinement of clinical care protocols (**Supplementary Table 10**).

### Validation of the diagnostic capability of ERBB3 for THCA and LNM potential

In an independent validation set (GSE60542), we noted significant differential expression of 11 of the 12 previously identified hub genes, with the exception of ANPEP, between normal thyroid tissue and thyroid tumors (**Supplementary Figure 6A**). We noted a significant upregulation of ERBB3 expression in thyroid tumors in both the validation set and TCGA-THCA cohort. Furthermore, the immunohistochemical analysis revealed a significant elevation in protein expression levels of ERBB3 in thyroid tumors compared to normal thyroid tissue (**Supplementary Figure 6B**). In the GSE60542 cohort, our ROC analysis demonstrated that ERBB3 exhibits excellent discriminatory power for thyroid tumors (AUC=0.89; **Supplementary Figure 6C**). Notably, our results indicate a significant upregulation in ERBB3 expression levels in metastatic lymph nodes compared to normal lymphoid tissue (**Supplementary Figure 6D**). ERBB3 also exhibited excellent diagnostic efficacy for metastatic lymph nodes (**Supplementary Figure 6E**).

### Exploration and validation of the therapeutic potential of ERBB3 in THCA

The subcellular localization of ERBB3 in tumor cells was investigated using ICC-IF and confocal microscopy techniques. ERBB3 was detected in the plasma membrane and actin filaments, and it is predicted to be secreted (**Supplementary Figures 7A, B**). The increased expression of ERBB3 in THCA, combined with its membrane localization, makes this protein an attractive target for cancer therapy.

**Figure 9:**
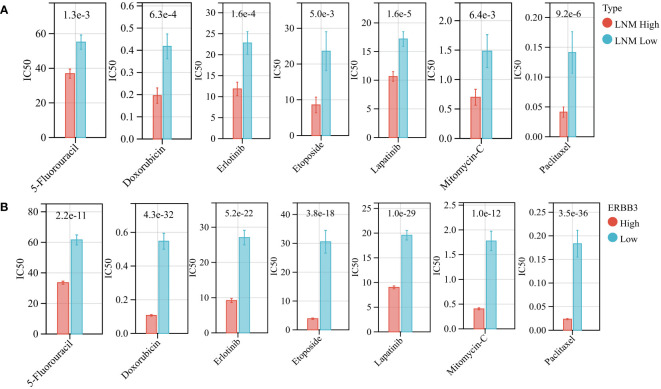
**(A)** The drug sensitivity of various anti-tumor drugs in patients with high and low LNM potential. **(B)** The drug sensitivity of various anti-tumor drugs in patients with high and low ERBB3 expression level.

Using the “oncoPredict” algorithm and the GDSC database, we evaluated the sensitivity of all tumor samples in TCGA-THCA to the anti-tumor drugs identified as potentially impacting LNM potential. Patients with high LNM potential and high expression of ERBB3 have lower half-maximal inhibitory concentrations (IC50) for Capecitabine, Doxorubicin, Epirubicin, Erlotinib Hydrochloride, Etoposide, Fluorouracil, Lapatinib, Mitomycin, and Paclitaxel, indicating increased sensitivity (**Figures 9A, B**).

**Figure 10:**
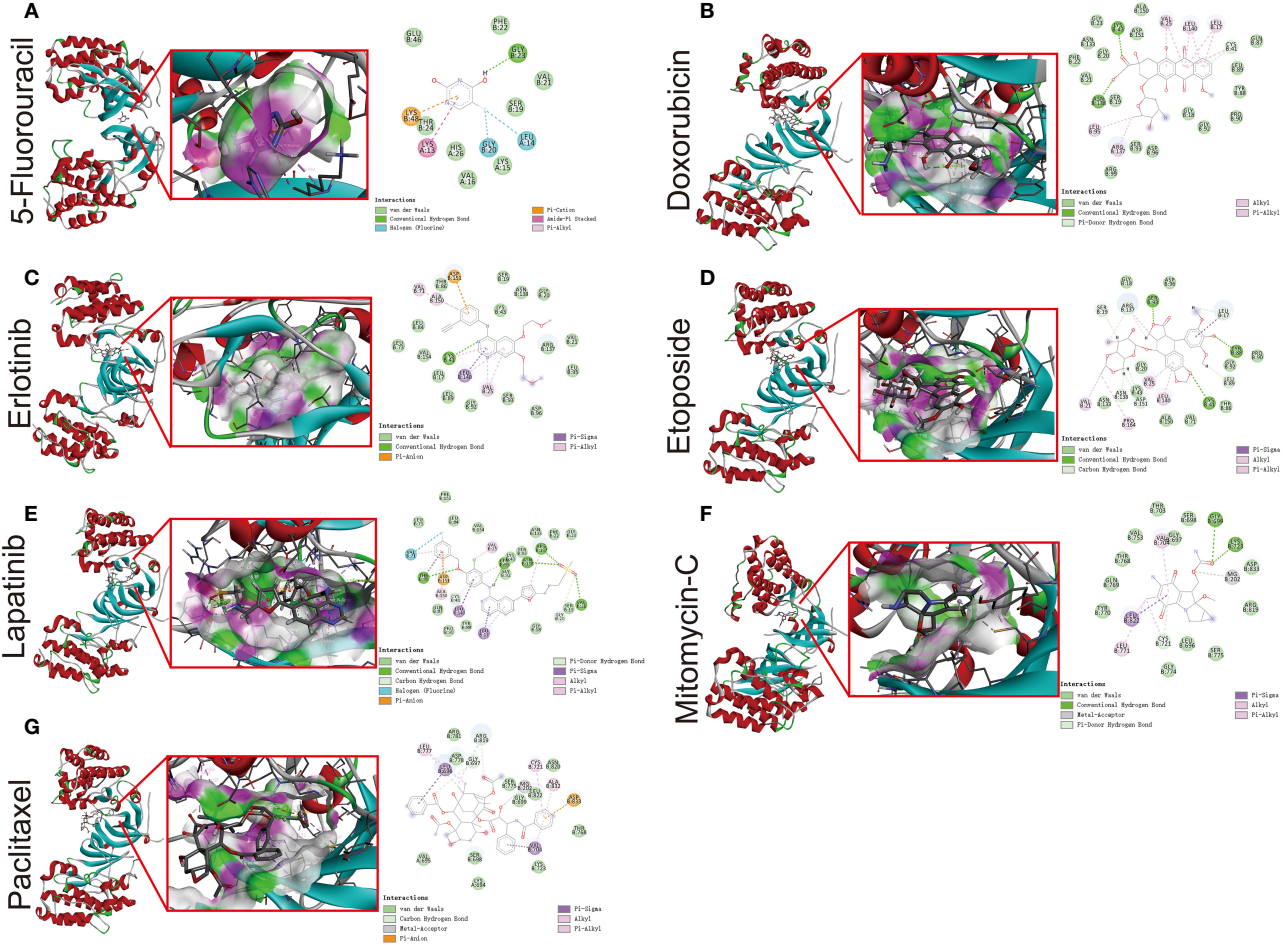
Molecular docking simulation between ERBB3 and 5-Fluorouracil **(A)**, Doxorubicin **(B)**, Erlotinib **(C)**, Etoposide **(D)**, Lapatinib **(E)**, Mitomycin-C **(F)**, and Paclitaxel **(G)**.

To further verify the strong correlation between ERBB3 and these potential therapeutic drugs, we performed molecular docking of these drugs with ERBB3. The three-dimensional and two-dimensional conformations of the molecular docking between Capecitabine, Doxorubicin, Epirubicin, Erlotinib Hydrochloride, Etoposide, Fluorouracil, Lapatinib, Mitomycin, and Paclitaxel with ERBB3 are shown in **Figures 10A–G**. The docking scores of Lapatinib, Etoposide, and Doxorubicin with ERBB3 are the most favorable, with values of -10.1 kcal/mol, -9.3 kcal/mol, and -8.8 kcal/mol, respectively.

We further conducted a meta-analysis to validate the therapeutic potential of Lapatinib in tumor patients with LNM. Since there is a scarcity of research studies on the therapeutic effects of Lapatinib in the treatment of thyroid cancer, we focused our investigation on endocrine-related tumors instead. A total of five clinical studies were collected (**Supplementary Figure 8A**) (67–70). The heterogeneity test result of the rates of achieving PCR between lapatinib combination therapy and monotherapy group was (Q=23.4, P=0.0001, I2 = 83%) and the combined value of the estimated effect was [RR=1.48, 95% CI (1.19, 1.86); P=0.0005]. The funnel plot presented is not suggestive of publication bias (**Supplementary Figure 8B**). Our meta-analysis indicates that the treatment regimen incorporating Lapatinib is more effective in achieving pathological complete response (PCR) in patients with LNM.

### Experimental validation of expression levels of ERBB3 in THCA cases with and without LNM

**Figure 11:**
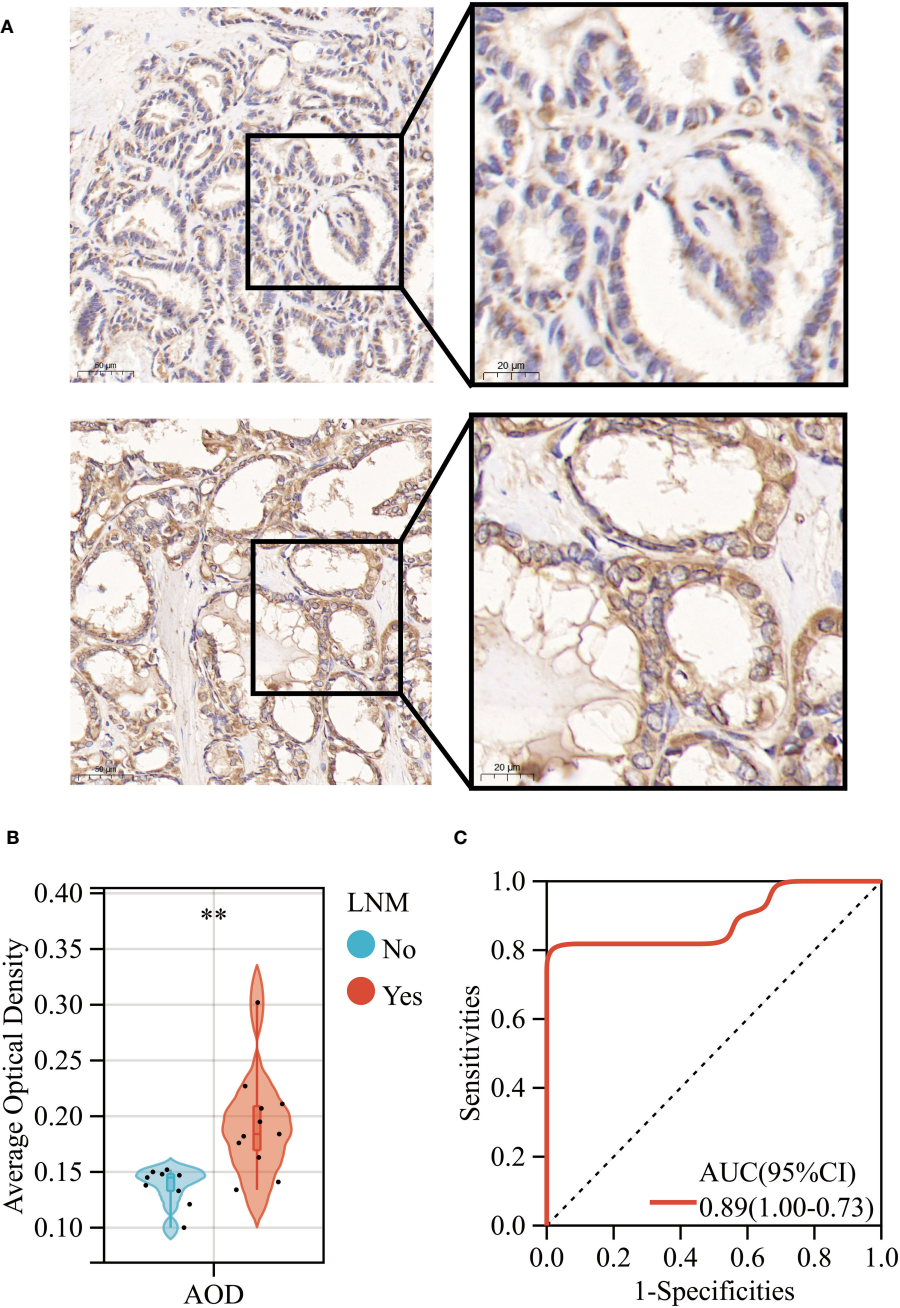
**(A)** Immunohistochemical expression levels of ERBB3 in THCA with (Lower) and without (Upper) lymph node metastasis. **(B)** AOD of ERBB3 protein immunohistochemical positive staining. **(C)** ROC curves of the AOD of ERBB3 protein for predicting LNM in THCA. **: *p*<0.01.

Primarily, we observed a significant upregulation in the gene expression levels of ERBB3 in THCA samples that had experienced LNM through RT-qPCR experimental analysis (**Supplementary Figure 9**). Subsequently, our IHC results revealed that while ERBB3 protein was expressed in the cytoplasm of THCA cases without LNM, a significant increase in the expression levels of the ERBB3 protein was evident in THCA cases with LNM (**Figure 11A**). This was also quantified by the AOD values measured for different pathological slides, thus corroborating the findings (**Figure 11B**). Moreover, the ROC analysis indicated that the AOD values of ERBB3 protein immunohistochemical positive staining could serve as a promising diagnostic biomarker for determining the occurrence of lymph node metastasis in THCA cases (AUC=0.89, 95%CI 0.73-1.00; **Figure 11C**).

## Discussion

LNM, particularly in the cervical region, is a common pathological feature encountered in THCA and may manifest in the early stages of the disease. In this study, we introduce a novel concept - LNM potential - aimed at elucidating the genetic basis of this phenomenon. Additionally, we employ a diverse range of bioinformatics analysis techniques, including WGCNA, machine learning, and molecular docking, to pinpoint the key gene underlying LNM potential and explore potentially therapeutic drugs targeting this gene.

Our study identified 12 hub genes as a potential high-risk biomarker for LNM in THCA. Simultaneously, we explored the association between the 12 hub genes and the biological processes and immune infiltration in THCA. Regardless of whether in THCA or pan-cancer, hub genes were significantly associated with the decrease of neutrophils and the increase of DC and macrophages in tumors. Considerable research has demonstrated the utility of neutrophil-to-lymphocyte ratio (NLR) in predicting lymph node metastasis in multiple types of cancer (71–74). The study conducted by Hiromu Fujita et al. revealed that the accumulation of neutrophils, especially CD16b-positive neutrophils, in the peritumoral region is an independent factor contributing to lymph node metastasis (75). Notably, the authors’ research was centered on thoracic esophageal squamous cell carcinoma (75). The investigations undertaken by Yuandong Liao et al. demonstrate that STC1-dependent immune escape from macrophage phagocytosis can be suppressed by the inhibition of competitive interaction between LNMAS and HMGB1, resulting in the abrogation of TWIST1 and STC1 chromatin accessibility, thereby suppressing cervical cancer lymph node metastasis (76). DC cells, as professional antigen-presenting cells, are responsible for presenting cancer-associated antigens to the adaptive immune system in the sentinel lymph nodes (77, 78). It has been observed that sentinel lymph nodes with macrometastases in cancer patients exhibit arrested maturation of dendritic cells, fewer interactions between mature dendritic cells and cytotoxic T cells, and an increased population of regulatory T cells, as opposed to sentinel lymph nodes without metastasis. However, these observations were not made when compared to healthy controls (79). Therefore, the physiological basis for the influence of hub genes on the lymph node metastatic potential of THCA lies in the observed differences in immune cell infiltration associated with these hub genes, particularly in neutrophils, DC cells, and macrophages. However, it is important to note that this study is based on bioinformatics techniques for estimating immune cell infiltration within tumors. Further in-depth experiments, such as flow cytometry and immunofluorescence, are required for the validation of clinical samples. Additionally, it’s worth mentioning that ITGA3, one of the 12 Hub genes we identified, has been found to serve as a biomarker of progression and recurrence in THCA (80). The results of the CCK-8 experiment conducted by Jizong Zhang et al. indicate that overexpression of ITGA3 significantly enhances the proliferation capability of thyroid cancer cell lines. Additionally, it markedly augments their invasive and migratory abilities (81).

It is worth noting that our pan-cancer analysis indicates a close correlation between the activation of these 12 hub genes and the oncological feature of EMT, a critical step in tumor invasion and metastasis (82). In particular, SNAI1 and FN1 were found to be positively correlated with EMT activation in more than half of the tumor types analyzed. Consistent with previous research, SNAI1 was identified as the first and most extensively studied transcription repressor of CDH1, a hallmark of EMT encoded by the epithelial gene encoding E-cadherin. Direct binding of SNAI1 to the E-boxes present in the CDH1 promoter leads to transcriptional repression of CDH1 expression (83). SNAI1 is an EMT regulatory factor that has been widely reported, which is consistent with our research findings. In cancer-associated EMT, SNAI1 serves as an imperative factor in driving the transition by strongly repressing E-cadherin and tight junction components (claudins), while also upregulating mesenchymal marker proteins, including vimentin and fibronectin (84). The study by Haihai Liang et al. revealed that knockdown of PTAL resulted in increased expression of miR-101 and consequent inhibition of FN1 expression, ultimately leading to upregulation of EMT, which in turn promoted the migration of OvCa cells (85). Thus, EMT represents another potential biological basis for the hub genes we have identified that can affect the LNM potential of THCA (86). In general, the identification of these hub genes provides a valuable and significant resource for further understanding and exploring the phenomenon of early LNM in THCA.

Furthermore, we have developed a nomogram capable of accurately predicting the likelihood of LNM in THCA patients. Additionally, we have established a web-based tool to access this nomogram’s prediction model. The nomogram presented in this study can be easily utilized in clinical practice through our web-based tool, offering valuable resources and guidance for the formulation of clinical treatment and care strategies for THCA patients (87, 88).

Subsequently, employing an integrative analysis of three machine learning techniques, we identify ERBB3 as the key gene influencing LNM potential. ErbB/HER receptor tyrosine kinases (RTKs) occupy a crucial position in animal development, and their dysfunctional operation may catalyze the pathophysiological progression of certain tumor types (89, 90). In mammals, the existence of four ErbB/HER receptors is expounded: the epidermal growth factor receptor (EGFR/HER1), HER2/ErbB2/neu, HER3/ErbB3, and HER4/ErbB4 (91). Physiological expression of these receptors has been reported in epithelial, mesenchymal, cardiac, and neuronal tissues. The gene ERBB3 codes for HER3, a discovery credited to Kraus et al. in 1989 (92). Located on human chromosome 12q13, HER3 exhibits a wide expression across adult human tissues, including cells from the reproductive, endocrine, urinary, gastrointestinal, respiratory, skin, and nervous systems (93–96). Structurally, HER3 comprises an extensive extracellular domain (ECD), an individual hydrophobic transmembrane segment, and an intracellular domain, which comprises a tyrosine-rich carboxyterminal tail, a juxtamembrane region, and a tyrosine kinase segment (97–99). Featuring four subdomains, the HER3 extracellular domain is known as subdomains I-IV. ERBB3 expression has been discovered to be upregulated in numerous types of tumors, including but not limited to breast, ovarian, lung, colon, pancreatic, melanoma, gastric, head and neck, and prostate cancers (100–105). In additional reports, targeting ERBB3, such as gene knockdown and knockout, has also been shown to impact the proliferation and migration of thyroid cancer. This implies that targeting ERBB3 may become one of the potential therapeutic targets for thyroid cancer (106). Notably, there exists limited research on ERBB3 in THCA, and at present, no studies have reported the potential biological functions of ERBB3 in THCA lymph node metastasis.

The gene expression level of ERBB3 has been found to be associated with distinct clinical characteristics of THCA, particularly the occurrence of LNM. Aberrant methylation of the gene promoter is a significant cause of deactivation (107–109). To further investigate the underlying mechanisms of ERBB3 gene expression alterations, our attention was directed towards the variation in methylation levels of ERBB3. Notably, clinical traits associated with upregulation of ERBB3 mRNA expression were always accompanied by decreased levels of ERBB3 methylation, and vice versa. Hence, the downregulation of ERBB3 gene expression is partly attributed to CpG island hypermethylation in its promoter region (104, 110).

THCA belongs to endocrine tumors which arise from specialized cells responsible for hormone secretion. The migration of tumor cells, which is a prerequisite for the development of metastasis, has been demonstrated to be controlled by signaling molecules in the environment, including neuroendocrine hormones (111–113). Therefore, our study investigated some potential EDCs that may impact the LNM potential of THCA in an ERBB3-dependent manner. Some of the discovered EDCs are substances that individuals may come into contact with in their daily lives, including Benzo(a)pyrene, bisphenol A, and copper; while others are drugs that may be used in the clinic, such as Estradiol, Tamoxifen, and Raloxifene Hydrochloride (114–119). Therefore, it may be necessary for THCA patients to avoid exposure to these substances or drugs in their daily lives.

We further discovered, via the CTD database, that 7 anti-tumor drugs have the potential to interact with ERBB3 and impact its gene expression levels (56). Subsequently, we utilized multiple techniques to validate these findings. Initially, the GDSC database indicated that ERBB3 serves as a biomarker for the sensitivity of these anti-tumor drugs (58). Further molecular docking validation revealed the binding affinity between these drugs and ERBB3 (120). Among these drugs, Lapatinib, Etoposide, and Doxorubicin displayed the strongest binding affinity with ERBB3, especially Lapatinib. Furthermore, our study suggests that THCA patients with high LNM potential may benefit more from Lapatinib, a finding that has not been previously documented in the literature. Additionally, we conducted a meta-analysis that demonstrated combination regimens containing Lapatinib to have better therapeutic efficacy for late-stage endocrine tumors with lymph node metastasis. Certainly, the physiological basis for targeting the ERBB3 protein is supported by its significant upregulation in THCA tumors and lymph nodes with metastasis (121). Additionally, subcellular structural analysis using immunofluorescence indicates that ERBB3 is primarily enriched on the cell membrane. It is well-known that more than 60% of all drug targets are membrane proteins, which is also one of the bases for ERBB3 to become a therapeutic target (122). Although no studies have been conducted in THCA, a randomized controlled study by Alexandra Leary et al. suggests that Lapatinib has antiproliferative effects in a subgroup of nonamplified breast tumors characterized by high HER3 expression. It is worth investigating the potential role of high HER2:HER3 heterodimers in predicting response to lapatinib (123). Very few studies have explored the role of lapatinib in thyroid cancer treatment. Koichi Ohno’s research discovered that the combined use of lapatinib and lenvatinib significantly inhibits the growth of TPC-1/LR (a drug-resistant thyroid cancer cell line) *in vitro* and in a xenograft mouse model (124). Lingxiao Cheng’s study suggests that the addition of lapatinib results in more pronounced changes in iodine and glucose regulation gene expression, sodium-iodine symporter membrane localization, radioactive iodine uptake, and cytotoxicity in thyroid cancer cells, indicating a more significant redifferentiation effect on thyroid cancer cells (125). Furthermore, due to the scarcity of reports about the role of lapatinib in thyroid cancer treatment, our investigation into lapatinib is also one of the novelty of this study. Therefore, our study proposes a potential therapeutic agent and target for THCA treatment, which requires further mechanism research to corroborate.

To validate the gene and protein expression levels of ERBB3 in THCA cases with or without LNM, we conducted RT-qPCR and IHC experiments. Encouragingly, our findings were consistent with the bioinformatic analysis we previously performed. Concurrently, we identified a quantitative index of IHC staining, AOD, which could serve as a diagnostic biomarker for determining the occurrence of lymph node metastasis in THCA cases. Remarkably, the AOD value exhibited satisfactory performance in the ROC analysis. Therefore, our IHC findings for ERBB3 in THCA cases indicate that it could serve as a useful auxiliary diagnostic tool. Furthermore, ERBB3 is significantly upregulated in lymph nodes that have undergone tumor metastasis compared to normal lymph nodes. Therefore, ERBB3 has the potential to assist pathologists in discriminating lymph nodes invaded by tumors. For LNM to occur, tumor cells must flow or settle in the marginal sinus of the lymph nodes (126–128). To detect cancer metastasis in the lymph nodes, pathologists need to search for tumor cells in the marginal sinus through multiple sections and tissue samples (129). However, confirming micro-metastases in some lymph nodes can be challenging (130). Therefore, determining whether lymph nodes have been invaded by tumors using ERBB3 as a marker could aid in the precise clinical staging of cancer.

This study has constructed several tools that can be further optimized and utilized in future clinical practice. Firstly, we have developed a novel molecular subtyping scheme that can preliminarily assess the tumor’s ability to develop LNM at the genetic level. Furthermore, we have built an online nomogram tool that can conveniently calculate the probability of LNM occurrence in different THCA patients based on our research. This tool can be used alongside the development of clinical treatment plans, taking into consideration the scores obtained from the online nomogram tool. In addition, our research has provided new evidence for future pathological precision diagnosis. Specifically, it suggests that ERBB3 positivity has the potential to assist in diagnosing lymph nodes that have already experienced THCA metastasis. This also presents a novel approach to confirming micro-metastases in some lymph nodes at the early stages of the disease.

In summary, we performed comprehensive analysis of THCA patients with different LNM potentials using multiple bioinformatic techniques. We explored the activities of different pathways and identified key genes that affect LNM potential. Additionally, we also screened potential therapeutic drugs and targets for THCA. Our study provides useful resources and new perspectives for the development and optimization of clinical treatment plans for THCA patients in the future. However, there are also limitations to our study. Firstly, although we utilized multiple datasets for exploration and validation, the lack of *in vivo* and *in vitro* experiments restricts the understanding of underlying mechanisms. Additionally, our study is akin to a retrospective analysis, and the conclusions drawn require further validation from prospective studies with larger sample sizes.

## Conclusion

Utilizing multiple bioinformatics analysis techniques, we have investigated differences in pathway activation and immune infiltration among THCA patients with varying LNM potential. Our analysis using WGCNA has revealed two gene modules that influence LNM potential, with a total of 12 genes identified as hub genes significantly impacting LNM potential. These hub genes primarily affect the infiltration levels of neutrophils, DC cells, and macrophages, as well as the activation of the EMT pathway in THCA. Employing multiple machine learning algorithms, we have identified ERBB3 as a key gene associated with LNM potential. We have observed that ERBB3 is upregulated in THCA patients with LNM and advanced THCA, and this upregulation may be attributed to changes in the methylation status of ERBB3. The interaction between ERBB3 and Lapatinib may present a potential therapeutic target for thyroid carcinoma patients who develop lymph node metastasis. Furthermore, we have developed a novel and user-friendly web-based tool (http://www.empowerstats.net/pmodel/?m=17617_LNM) that utilizes a nomogram to assess the potential for LNM in THCA patients. Our study lays the foundation for future investigations into the underlying mechanisms driving differences in lymph node metastatic potential among cases of thyroid carcinoma. Therefore, our findings provide valuable resources and guidance for the development of personalized clinical treatment plans for patients with this disease.

## Data availability statement

Publicly available datasets were analyzed in this study. This data can be found here: TCGA (https://portal.gdc.cancer.gov/projects/TCGA-THCA) and GEO database (https://www.ncbi.nlm.nih.gov/geo/).

## Ethics statement

The studies involving human participants were reviewed and approved by the Ethics Committees of the Third Affiliated Hospital of Anhui Medical University. The patients/participants provided their written informed consent to participate in this study. Written informed consent was obtained from the individual(s) for the publication of any potentially identifiable images or data included in this article.

## Author contributions

Conceptualization, YL. Data curation, ZY. Formal analysis, YL. Investigation, YL, WY and HC. Methodology, YL. Resources, ZY. Supervision, HC. Validation, WY and HC. Writing – original draft, YL and WY. Writing – review & editing, HC. All authors contributed to the article and approved the submitted version.

## Supplementary material

Supplementary Figure 1 Enrichment analysis results for up- **(A)** and down-regulated **(B)** genes in THCA. Distinctive patterns in immune infiltration **(C)** and cancer-related pathway activation **(D)** in THCA patients, stratified by high or low LNM potentials.

Supplementary Figure 2
**(A)** Chromosomal locations of DEGs in normal thyroid and THCA. **(B)** A heatmap of inter-module distances between different gene modules. Correlation between module membership (MM) and gene significance (GS) for blue **(C)**, pink **(D)**, black **(E)**, purple **(F)**, magenta **(G)**, and green **(H)** gene modules.

Supplementary Figure 3 Sample was classified as either CNV-Amp, CNV-Del, or SNV-Mutant based on the occurrence of a CNV or SNV alteration in at least one of the identified LNM potential-related hub genes. A volcano plot reveals significant differences in immune cell infiltration between THCA patients with CNV **(A)** and SNV alterations **(B)** relative to the wild-type group. **(C)** Assessing the correlation between the GSVA scores of LNM potential-related hub genes and immune cell infiltrate levels in types of cancer, including THCA **(D)**, using Spearman’s correlation analysis. **(E)** Summary of the correlations between GSVA score and cancer-related pathway activity among 33 cancer types. (*: *p* value <= 0.05; #: FDR <= 0.05).

Supplementary Figure 4 Expression of ERBB3 in THCA patients differentiated by gender **(A)**, site of occurrence **(B)**, number of primary tumors **(C)**, and extent of surgical resection **(D)**.

Supplementary Figure 5
**(A)** Methylation levels of ERBB3 in tumor specimens compared to their corresponding normal tissues among 33 cancer types. The position of CpG islands **(B)** and CpG sites **(C)** of ERBB3 used for DNA methylation analyses. Methylation levels of ERBB3 in THCA patients with different LNM potential **(D)**, age **(E)**, gender **(F)**, N **(G)**/M **(H)**/T **(I)** staging, tumor stage **(J)**, tumor location **(K)**, number of primary tumors (L), extent of surgical resection **(M)**, and history of thyroid gland disorder **(N)**.

Supplementary Figure 6
**(A)** Expression levels of LNM potential related-hub genes in primary THCA and normal thyroid tissues in the GSE60542 cohort. **(B)** Protein expression levels of ERBB3 in primary THCA and normal thyroid tissues. **(C)** ROC analysis of ERBB3 for diagnosis in primary THCA and normal thyroid tissues in the GSE60542 cohort. **(D)** Gene expression levels of ERBB3 in normal lymph nodes and lymph nodes with metastatic tumors. **(E)** ROC analysis of ERBB3 for diagnosis in lymph nodes with metastatic tumors.

Supplementary Figure 7
**(A)** Subcellular localization of ERBB3 protein in various tumor cells. **(B)** Schematic representation of subcellular localization of ERBB3 protein in tumor cells.

Supplementary Figure 8
**(A)** Meta-analysis validates the efficacy of combined treatment regimen with Lapatinib in advanced endocrine organ tumors with lymph node metastasis. **(B)** Assessment of publication bias via funnel plot analysis.

Supplementary Figure 9 qRT-PCR verification of expression of ERBB3 in THCA with (Yes) and without (No) lymph node metastasis (***: *p<*0.001).

## References

[1] Sanabria A Kowalski LP Shah JP Nixon IJ Angelos P Williams MD. Growing incidence of thyroid carcinoma in recent years: Factors underlying overdiagnosis. Head Neck (2018) 40(4):855–66. doi: 10.1002/hed.25029 PMC5849517 29206325

[2] LiVolsi VA. Papillary thyroid carcinoma: an update. Mod Pathol (2011) 24 Suppl 2:S1–9. doi: 10.1038/modpathol.2010.129 21455196

[3] Jensen K Patel A Hoperia V Larin A Bauer A Vasko V. Dynamic changes in E-cadherin gene promoter methylation during metastatic progression in papillary thyroid cancer. Exp Ther Med (2010) 1(3):457–62. doi: 10.3892/etm_00000071 PMC3445881 22993562

[4] Zhang LY Chen Y Ao YZ. Value of thyroglobulin combined with ultrasound-guided fine-needle aspiration cytology for diagnosis of lymph node metastasis of thyroid carcinoma. World J Clin Cases (2022) 10(2):492–501. doi: 10.12998/wjcc.v10.i2.492 35097074 PMC8771387

[5] Mao J Zhang Q Zhang H Zheng K Wang R Wang G. Risk factors for lymph node metastasis in papillary thyroid carcinoma: A systematic review and meta-analysis. Front Endocrinol (Lausanne) (2020) 11:265. doi: 10.3389/fendo.2020.00265 32477264 PMC7242632

[6] Lo CY. Lymph node dissection for papillary thyroid carcinoma. Methods Mol Biol (2022) 2534:57–78. doi: 10.1007/978-1-0716-2505-7_5 35670968

[7] He Y Pan MZ Huang JM Xie P Zhang F Wei LG. Iodine-131: an effective method for treating lymph node metastases of differentiated thyroid cancer. Med Sci Monit (2016) 22:4924–8. doi: 10.12659/MSM.899028 PMC5181522 27974741

[8] Czarniecka A Jarzab M Krajewska J Chmielik E Szcześniak-Klusek B Stobiecka E. Prognostic value of lymph node metastases of differentiated thyroid cancer (DTC) according to the local advancement and range of surgical excision. Thyroid Res (2010) 3:8. doi: 10.1186/1756-6614-3-8 21034453 PMC2987863

[9] Pino A Mazzeo C Frattini F Zhang D Wu CW Zanghì G. Lymph node dissection morbidity in thyroid cancer: an integrative review. Sisli Etfal Hastan Tip Bul (2021) 55:433–7. doi: 10.14744/SEMB.2021.33401 PMC8907691 35317379

[10] Liu C Xiao C Chen J Li X Feng Z Gao Q. Risk factor analysis for predicting cervical lymph node metastasis in papillary thyroid carcinoma: a study of 966 patients. BMC Cancer (2019) 19(1):622. doi: 10.1186/s12885-019-5835-6 31238891 PMC6593593

[11] Wang Y Deng C Shu X Yu P Wang H Su X. Risk factors and a prediction model of lateral lymph node metastasis in CN0 papillary thyroid carcinoma patients with 1-2 central lymph node metastases. Front Endocrinol (Lausanne) (2021) 12:716728. doi: 10.3389/fendo.2021.716728 34721289 PMC8555630

[12] Guang Y He W Zhang W Zhang H Zhang Y Wan F. Clinical study of ultrasonographic risk factors for central lymph node metastasis of papillary thyroid carcinoma. Front Endocrinol (Lausanne) (2021) 12:791970. doi: 10.3389/fendo.2021.791970 34917039 PMC8669800

[13] Ruiz EML Niu T Zerfaoui M Kunnimalaiyaan M Friedlander PL Abdel-Mageed AB. A novel gene panel for prediction of lymph-node metastasis and recurrence in patients with thyroid cancer. Surgery (2020) 167(1):73–9. doi: 10.1016/j.surg.2019.06.058 31711617

[14] Zhang Q Li J Shen H Bai X Zhang T Liu P. Screening and validation of lymph node metastasis risk-factor genes in papillary thyroid carcinoma. Front Endocrinol (Lausanne) (2022) 13:991906. doi: 10.3389/fendo.2022.991906 36465624 PMC9714616

[15] Huang C Su X Zhou DL Xu BH Liu Q Zhang X. A diagnostic and predictive lncRNA lnc-MPEG1-1 promotes the proliferation and metastasis of papillary thyroid cancer cells by occupying miR-766-5p. Mol Ther Nucleic Acids (2022) 28:408–22. doi: 10.1016/j.omtn.2022.03.023 PMC9036069 35505969

[16] Wei M Wang R Zhang W Zhang J Fang Q Fang Z. Landscape of gene mutation in Chinese thyroid cancer patients: Construction and validation of lymph node metastasis prediction model based on clinical features and gene mutation marker. Cancer Med (2023) 12(11):12929–42. doi: 10.1002/cam4.5945 PMC10278465 37081757

[17] Feng Y Min Y Chen H Xiang K Wang X Yin G. Construction and validation of a nomogram for predicting cervical lymph node metastasis in classic papillary thyroid carcinoma. J Endocrinol Invest (2021) 44(10):2203–11. doi: 10.1007/s40618-021-01524-5 33586026

[18] Gao L Li X Xia Y Liu R Liu C Shi X. Large-volume lateral lymph node metastasis predicts worse prognosis in papillary thyroid carcinoma patients with N1b. Front Endocrinol (Lausanne) (2021) 12:815207. doi: 10.3389/fendo.2021.815207 35185788 PMC8847215

[19] Alwadi D Felty Q Yoo C Roy D Deoraj A. Endocrine disrupting chemicals influence hub genes associated with aggressive prostate cancer. Int J Mol Sci (2023) 24(4):3191. doi: 10.3390/ijms24043191 36834602 PMC9959535

[20] Yilmaz B Terekeci H Sandal S Kelestimur F. Endocrine disrupting chemicals: exposure, effects on human health, mechanism of action, models for testing and strategies for prevention. Rev Endocr Metab Disord (2020) 21(1):127–47. doi: 10.1007/s11154-019-09521-z 31792807

[21] Kim H Kim HS Piao YJ Moon WK. Bisphenol A promotes the invasive and metastatic potential of ductal carcinoma *in situ* and protumorigenic polarization of macrophages. Toxicol Sci (2019) 170(2):283–95. doi: 10.1093/toxsci/kfz119 31143956

[22] Jordan VC. Fourteenth Gaddum Memorial Lecture. A current view of tamoxifen for the treatment and prevention of breast cancer. Br J Pharmacol (1993) 110(2):507–17. 10.1111/j.1476-5381.1993.tb13840.x PMC2175926 8242225

[23] Alsen M Sinclair C Cooke P Ziadkhanpour K Genden E van Gerwen M. Endocrine disrupting chemicals and thyroid cancer: an overview. Toxics (2021) 9:14. doi: 10.3390/toxics9010014 33477829 PMC7832870

[24] Li L Ying Y Zhang C Wang W Li Y Feng Y. Bisphenol A exposure and risk of thyroid nodules in Chinese women: A case-control study. Environ Int (2019) 126:321–8. doi: 10.1016/j.envint.2019.02.026 30825751

[25] Boas M Feldt-Rasmussen U Main KM. Thyroid effects of endocrine disrupting chemicals. Mol Cell Endocrinol (2012) 355:240–8. doi: 10.1016/j.mce.2011.09.005 21939731

[26] Reuter JA Spacek DV Snyder MP. High-throughput sequencing technologies. Mol Cell (2015) 58(4):586–97. doi: 10.1016/j.molcel.2015.05.004 PMC4494749 26000844

[27] Wajnberg G Passetti F. Using high-throughput sequencing transcriptome data for INDEL detection: challenges for cancer drug discovery. Expert Opin Drug Discov (2016) 11(3):257–68. doi: 10.1517/17460441.2016.1143813 26787005

[28] Xu J Liao K Yang X Wu C Wu W. Using single-cell sequencing technology to detect circulating tumor cells in solid tumors. Mol Cancer (2021) 20(1):104. doi: 10.1186/s12943-021-01392-w 34412644 PMC8375060

[29] Chen H Liu Y Yin Z Chen H Wang Y Qian Y. Homologous repair deficiency-associated genes in invasive breast cancer revealed by WGCNA co-expression network analysis and genetic perturbation similarity analysis. Cell Cycle (2023) 22(9):1077–100. doi: 10.1080/15384101.2023.2174339 PMC10081085 36757135

[30] Chen H Zhang J Sun X Wang Y Qian Y. Mitophagy-mediated molecular subtypes depict the hallmarks of the tumor metabolism and guide precision chemotherapy in pancreatic adenocarcinoma. Front Cell Dev Biol (2022) 10:901207. doi: 10.3389/fcell.2022.901207 35938160 PMC9353335

[31] Wang Y Sun J Yang Y Zebaze Dongmo S Qian Y Wang Z. Identification and development of subtypes with poor prognosis in gastric cancer based on both hypoxia and immune cell infiltration. Int J Gen Med (2021) 14:9379–99. doi: 10.2147/IJGM.S326647 PMC8664384 34908867

[32] Wang Y Wang Z Sun J Qian Y. Identification of HCC subtypes with different prognosis and metabolic patterns based on mitophagy. Front Cell Dev Biol (2021) 9:799507. doi: 10.3389/fcell.2021.799507 34977039 PMC8716756

[33] Tomczak K Czerwińska P Wiznerowicz M. The Cancer Genome Atlas (TCGA): an immeasurable source of knowledge. Contemp Oncol (Pozn) (2015) 19(1A):A68–77. doi: 10.5114/wo.2014.47136 PMC4322527 25691825

[34] Clough E Barrett T. The gene expression omnibus database. Methods Mol Biol (2016) 1418:93–110. doi: 10.1007/978-1-4939-3578-9_5 27008011 PMC4944384

[35] Tang Z Li C Kang B Gao G Li C Zhang Z. GEPIA: a web server for cancer and normal gene expression profiling and interactive analyses. Nucleic Acids Res (2017) 45(W1):W98–W102. doi: 10.1093/nar/gkx247 28407145 PMC5570223

[36] Langfelder P Horvath S. WGCNA: an R package for weighted correlation network analysis. BMC Bioinf (2008) 9:559. doi: 10.1186/1471-2105-9-559 PMC2631488 19114008

[37] Szklarczyk D Gable AL Nastou KC Lyon D Kirsch R Pyysalo S. The STRING database in 2021: customizable protein-protein networks, and functional characterization of user-uploaded gene/measurement sets. Nucleic Acids Res (2021) 49(D1):D605–12. doi: 10.1093/nar/gkaa1074 PMC7779004 33237311

[38] Tang Y Li M Wang J Pan Y Wu FX. CytoNCA: a cytoscape plugin for centrality analysis and evaluation of protein interaction networks. Biosystems (2015) 127:67–72. doi: 10.1016/j.biosystems.2014.11.005 25451770

[39] Zhou G Soufan O Ewald J Hancock R Basu N Xia J. NetworkAnalyst 3.0: a visual analytics platform for comprehensive gene expression profiling and meta-analysis. Nucleic Acids Res (2019) 47(W1):W234–41. doi: 10.1093/nar/gkz240 PMC6602507 30931480

[40] Jou J Gabdank I Luo Y Lin K Sud P Myers Z. The ENCODE portal as an epigenomics resource. Curr Protoc Bioinf (2019) 68(1):e89. doi: 10.1002/cpbi.89 PMC7307447 31751002

[41] Huang HY Lin YC Li J Huang KY Shrestha S Hong HC. miRTarBase 2020: updates to the experimentally validated microRNA-target interaction database. Nucleic Acids Res (2020) 48(D1):D148–54. doi: 10.1093/nar/gkz896 PMC7145596 31647101

[42] Liu ZP Wu C Miao H Wu H. RegNetwork: an integrated database of transcriptional and post-transcriptional regulatory networks in human and mouse. Database (Oxford) (2015) 2015:bav095. doi: 10.1093/database/bav095 26424082 PMC4589691

[43] Zhou Y Zhou B Pache L Chang M Khodabakhshi AH Tanaseichuk O. Metascape provides a biologist-oriented resource for the analysis of systems-level datasets. Nat Commun (2019) 10(1):1523. doi: 10.1038/s41467-019-09234-6 30944313 PMC6447622

[44] Hänzelmann S Castelo R Guinney J. GSVA: gene set variation analysis for microarray and RNA-seq data. BMC Bioinf (2013) 14:7. doi: 10.1186/1471-2105-14-7 PMC3618321 23323831

[45] Chen MM Li J Wang Y Akbani R Lu Y Mills GB. TCPA v3.0: an integrative platform to explore the pan-cancer analysis of functional proteomic data. Mol Cell Proteomics (2019) 18(8 suppl 1):S15–25. doi: 10.1074/mcp.RA118.001260 PMC6692772 31201206

[46] Labrie M Fang Y Kendsersky ND Li J Liang H Westin SN. Using reverse phase protein array (RPPA) to identify and target adaptive resistance. Adv Exp Med Biol (2019) 1188:251–66. doi: 10.1007/978-981-32-9755-5_14 PMC7382818 31820393

[47] Liu CJ Hu FF Xie GY Miao YR Li XW Zeng Y. GSCA: an integrated platform for gene set cancer analysis at genomic, pharmacogenomic and immunogenomic levels. Brief Bioinform (2023) 24(1):bbac558. doi: 10.1093/bib/bbac558 36549921

[48] Miao YR Zhang Q Lei Q Luo M Xie GY Wang H. ImmuCellAI: A unique method for comprehensive T-cell subsets abundance prediction and its application in cancer immunotherapy. Adv Sci (Weinh) (2020) 7(7):1902880. doi: 10.1002/advs.201902880 32274301 PMC7141005

[49] Wilkerson MD Hayes DN. ConsensusClusterPlus: a class discovery tool with confidence assessments and item tracking. Bioinformatics (2010) 26(12):1572–3. doi: 10.1093/bioinformatics/btq170 PMC2881355 20427518

[50] Kang J Choi YJ Kim IK Lee HS Kim H Baik SH. LASSO-based machine learning algorithm for prediction of lymph node metastasis in T1 colorectal cancer. Cancer Res Treat (2021) 53(3):773–83. doi: 10.4143/crt.2020.974 PMC8291173 33421980

[51] Feng Y Yan X. Support vector machine based lane-changing behavior recognition and lateral trajectory prediction. Comput Intell Neurosci (2022) 2022:3632333. doi: 10.1155/2022/3632333 35592714 PMC9113884

[52] Rigatti SJ. Random forest. J Insur Med (2017) 47(1):31–9. doi: 10.17849/insm-47-01-31-39.1 28836909

[53] Engebretsen S Bohlin J. Statistical predictions with glmnet. Clin Epigenetics (2019) 11(1):123. doi: 10.1186/s13148-019-0730-1 31443682 PMC6708235

[54] Xu N Guo H Li X Zhao Q Li J. A five-genes based diagnostic signature for sepsis-induced ARDS. Pathol Oncol Res (2021) 27:580801. doi: 10.3389/pore.2021.580801 34393665 PMC8357742

[55] Li S Que Y Yang R He P Xu S Hu Y. Construction of osteosarcoma diagnosis model by random forest and artificial neural network. J Pers Med (2023) 13(3):447. doi: 10.3390/jpm13030447 36983630 PMC10056981

[56] Davis AP Grondin CJ Johnson RJ Sciaky D Wiegers J Wiegers TC. Comparative toxicogenomics database (CTD): update 2021. Nucleic Acids Res (2021) 49(D1):D1138–43. doi: 10.1093/nar/gkaa891 PMC7779006 33068428

[57] Maeser D Gruener RF Huang RS. oncoPredict: an R package for predicting *in vivo* or cancer patient drug response and biomarkers from cell line screening data. Brief Bioinform (2021) 22(6):bbab260. doi: 10.1093/bib/bbab260 34260682 PMC8574972

[58] Yang W Soares J Greninger P Edelman EJ Lightfoot H Forbes S. Genomics of Drug Sensitivity in Cancer (GDSC): a resource for therapeutic biomarker discovery in cancer cells. Nucleic Acids Res (2013) 41(Database issue):D955–61. doi: 10.1093/nar/gks1111 PMC3531057 23180760

[59] Burley SK Berman HM Kleywegt GJ Markley JL Nakamura H Velankar S. Protein data bank (PDB): the single global macromolecular structure archive. Methods Mol Biol (2017) 1607:627–41. doi: 10.1007/978-1-4939-7000-1_26 PMC5823500 28573592

[60] Kim S Chen J Cheng T Gindulyte A He J He S. PubChem in 2021: new data content and improved web interfaces. Nucleic Acids Res (2021) 49(D1):D1388–95. doi: 10.1093/nar/gkaa971 PMC7778930 33151290

[61] Odhar HA Rayshan AM Ahjel SW Hashim AA Albeer A. Molecular docking enabled updated screening of the matrix protein VP40 from Ebola virus with millions of compounds in the MCULE database for potential inhibitors. Bioinformation (2019) 15(9):627–32. doi: 10.6026/97320630015627 PMC6859706 31787811

[62] Zheng Q Min S Zhou Q. Identification of potential diagnostic and prognostic biomarkers for LUAD based on TCGA and GEO databases. Biosci Rep (2021) 41(6):BSR20204370. doi: 10.1042/BSR20204370 34017995 PMC8182989

[63] Hu Y Li X Wang L Han B Nie S. T-distribution stochastic neighbor embedding for fine brain functional parcellation on rs-fMRI. Brain Res Bull (2020) 162:199–207. doi: 10.1016/j.brainresbull.2020.06.007 32603775

[64] Lopes AM Tenreiro MaChado JA. Uniform manifold approximation and projection analysis of soccer players. Entropy (Basel) (2021) 23(7):793. doi: 10.3390/e23070793 34201479 PMC8307339

[65] Ben Salem K Ben Abdelaziz A. Principal component analysis (PCA). Tunis Med (2021) 99(4):383–9. PMC8734479 35244921

[66] Ye Y Xiang Y Ozguc FM Kim Y Liu CJ Park PK. The genomic landscape and pharmacogenomic interactions of clock genes in cancer chronotherapy. Cell Syst (2018) 6:314–28.e2. doi: 10.1016/j.cels.2018.01.013 29525205 PMC6056007

[67] Baselga J Bradbury I Eidtmann H Di Cosimo S de Azambuja E Aura C. Lapatinib with trastuzumab for HER2-positive early breast cancer (NeoALTTO): a randomized, open-label, multicenter, phase 3 trial. Lancet (2012) 379(9816):633–40. doi: 10.1016/S0140-6736(11)61847-3 PMC5705192 22257673

[68] Guarneri V Frassoldati A Bottini A Cagossi K Bisagni G Sarti S. Preoperative chemotherapy plus trastuzumab, lapatinib, or both in human epidermal growth factor receptor 2-positive operable breast cancer: results of the randomized phase II CHER-LOB study. J Clin Oncol (2012) 30(16):1989–95. doi: 10.1200/JCO.2011.39.0823 22493419

[69] Robidoux A Tang G Rastogi P Geyer CE Jr Azar CA Atkins JN. Lapatinib as a component of neoadjuvant therapy for HER2-positive operable breast cancer (NSABP protocol B-41): an open-label, randomised phase 3 trial. Lancet Oncol (2013) 14(12):1183–92. doi: 10.1016/S1470-2045(13)70411-X 24095300

[70] Carey LA Berry DA Cirrincione CT Barry WT Pitcher BN Harris LN. Molecular heterogeneity and response to neoadjuvant human epidermal growth factor receptor 2 targeting in CALGB 40601, a randomized phase III trial of paclitaxel plus trastuzumab with or without lapatinib. J Clin Oncol (2016) 34(6):542–9. doi: 10.1200/JCO.2015.62.1268 PMC4980567 26527775

[71] Wang B Liu J Zhong Z. Prediction of lymph node metastasis in oral tongue squamous cell carcinoma using the neutrophil-to-lymphocyte ratio and platelet-to-neutrophil ratio. J Clin Lab Anal (2021) 35(6):e23684. doi: 10.1002/jcla.23684 33942387 PMC8183927

[72] Urs AB Augustine J Khurana N Uniyal A Passey JC Meher R. Preoperative platelet-lymphocyte ratio and neutrophil-lymphocyte ratio as predictors of occult lymph node metastasis detected using Desmoglein 3 and Cytokeratin in Indian population. J Oral Maxillofac Pathol (2022) 26(4):596. doi: 10.4103/jomfp.jomfp_49_21 37082044 PMC10112119

[73] Xia X Li K Wu R Lv Q Deng X Fei Z. Predictive value of neuron-specific enolase, neutrophil-to-lymphocyte-ratio and lymph node metastasis for distant metastasis in small cell lung cancer. Clin Respir J (2020) 14(11):1060–6. doi: 10.1111/crj.13242 32750207

[74] Song CY Meng YL Liu B Yan L Shang PZ Jia ZF. [Correlation analysis of neutrophil-to-lymphocyte ratio and platelet-to-lymphocyte ratio and central cervical lymph node metastasis of papillary thyroid microcarcinoma]. Zhonghua Zhong Liu Za Zhi (2021) 43(9):944–8. doi: 10.3760/cma.j.cn112152-20200509-00434 34530577

[75] Fujita H Motoyama S An J Nagakai Y Yamaguchi T Koyota S. Peritumoral CD16b positive-neutrophil accumulation strongly correlates with regional lymph node metastasis in thoracic esophageal squamous cell cancer. Surgery (2022) 171(6):1535–42. doi: 10.1016/j.surg.2021.11.022 35000783

[76] Liao Y Huang J Liu P Zhang C Liu J Xia M. Downregulation of LNMAS orchestrates partial EMT and immune escape from macrophage phagocytosis to promote lymph node metastasis of cervical cancer. Oncogene (2022) 41(13):1931–43. doi: 10.1038/s41388-022-02202-3 PMC8956512 35152264

[77] Yin X Chen S Eisenbarth SC. Dendritic cell regulation of T helper cells. Annu Rev Immunol (2021) 39:759–90. doi: 10.1146/annurev-immunol-101819-025146 33710920

[78] Waisman A Lukas D Clausen BE Yogev N. Dendritic cells as gatekeepers of tolerance. Semin Immunopathol (2017) 39(2):153–63. doi: 10.1007/s00281-016-0583-z 27456849

[79] Mansfield AS Heikkila P von Smitten K Vakkila J Leidenius M. Metastasis to sentinel lymph nodes in breast cancer is associated with maturation arrest of dendritic cells and poor co-localization of dendritic cells and CD8+ T cells. Virchows Arch (2011) 459(4):391–8. doi: 10.1007/s00428-011-1145-3 21894561

[80] Zhang G Li B Lin Y. Evaluation of ITGA3 as a biomarker of progression and recurrence in papillary thyroid carcinoma. Front Oncol (2021) 11:614955. doi: 10.3389/fonc.2021.614955 35174063 PMC8841514

[81] Zhang J Zhong Y Sang Y Ren G. miRNA-144-5p/ITGA3 suppressed the tumor-promoting behaviors of thyroid cancer cells by downregulating ITGA3. Comput Math Methods Med (2021) 2021:9181941. doi: 10.1155/2021/9181941 34938358 PMC8687768

[82] Bakir B Chiarella AM Pitarresi JR Rustgi AK. EMT. MET, plasticity, and tumor metastasis. Trends Cell Biol (2020) 30(10):764–76. doi: 10.1016/j.tcb.2020.07.003 PMC7647095 32800658

[83] Dong B Wu Y. Epigenetic regulation and post-translational modifications of SNAI1 in cancer metastasis. Int J Mol Sci (2021) 22(20):11062. doi: 10.3390/ijms222011062 34681726 PMC8538584

[84] Sundararajan V Tan M Zea Tan T Pang QY Ye J Chung VY. SNAI1-driven sequential EMT changes attributed by selective chromatin enrichment of RAD21 and GRHL2. Cancers (Basel) (2020) 12:1140. doi: 10.3390/cancers12051140 32370157 PMC7281482

[85] Liang H Yu M Yang R Zhang L Zhang L Zhu D. A PTAL-miR-101-FN1 axis promotes EMT and invasion-metastasis in serous ovarian cancer. Mol Ther Oncolytics (2020) 16:53–62. doi: 10.1016/j.omto.2019.12.002 31930166 PMC6951825

[86] Aiello NM Kang Y. Context-dependent EMT programs in cancer metastasis. J Exp Med (2019) 216(5):1016–26. doi: 10.1084/jem.20181827 PMC6504222 30975895

[87] Wu Z Zou Y Fu R Jin P Yuan H. A nomogram for predicting sclerotherapy response for treatment of lymphatic malformations in children. Eur J Med Res (2022) 27(1):209. doi: 10.1186/s40001-022-00844-3 36271467 PMC9585839

[88] Zhu H Gao Y Wang C Chen Z Yu X Qi X. A nomogram for decision-making assistance on surgical treatment of chronic osteomyelitis in long bones: Establishment and validation based on a retrospective multicenter cohort. Int J Surg (2022) 99:106267. doi: 10.1016/j.ijsu.2022.106267 35202861

[89] Wang Z. ErbB receptors and cancer. Methods Mol Biol (2017) 1652:3–35. doi: 10.1007/978-1-4939-7219-7_1 28791631

[90] Arndt- Jovin DJ Botelho MG Jovin TM. Structure-function relationships of ErbB RTKs in the plasma membrane of living cells. Cold Spring Harb Perspect Biol (2014) 6(4):a008961. doi: 10.1101/cshperspect.a008961 24691959 PMC3970415

[91] Patnaik SK Chandrasekar M Nagarjuna P Ramamurthi D Swaroop AK. Targeting of erbB1, erbB2, and their dual targeting using small molecules and natural peptides: blocking EGFR cell signaling pathways in cancer: A mini-review. Mini Rev Med Chem (2022) 22(22):2831–46. doi: 10.2174/1389557522666220512152448 35549881

[92] Kraus MH Issing W Miki T Popescu NC Aaronson SA. Isolation and characterization of ERBB3, a third member of the ERBB/epidermal growth factor receptor family: evidence for overexpression in a subset of human mammary tumors. Proc Natl Acad Sci USA (1989) 86(23):9193–7. doi: 10.1073/pnas.86.23.9193 PMC298460 2687875

[93] Gandullo- Sánchez L Ocaña A Pandiella A. HER3 in cancer: from the bench to the bedside. J Exp Clin Cancer Res (2022) 41(1):310. doi: 10.1186/s13046-022-02515-x 36271429 PMC9585794

[94] Haikala HM Jänne PA. Thirty years of HER3: from basic biology to therapeutic interventions. Clin Cancer Res (2021) 27(13):3528–39. doi: 10.1158/1078-0432.CCR-20-4465 PMC8254743 33608318

[95] Gil V Miranda S Riisnaes R Gurel B D'Ambrosio M Vasciaveo A. HER3 is an actionable target in advanced prostate cancer. Cancer Res (2021) 81(24):6207–18. doi: 10.1158/0008-5472.CAN-21-3360 PMC8932336 34753775

[96] Yonesaka K Tanizaki J Maenishi O Haratani K Kawakami H Tanaka K. HER3 augmentation via blockade of EGFR/AKT signaling enhances anticancer activity of HER3-targeting patritumab deruxtecan in EGFR-mutated non-small cell lung cancer. Clin Cancer Res (2022) 28(2):390–403. doi: 10.1158/1078-0432.CCR-21-3359 34921025

[97] Diwanji D Trenker R Thaker TM Wang F Agard DA Verba KA. Structures of the HER2-HER3-NRG1β complex reveal a dynamic dimer interface. Nature (2021) 600(7888):339–43. doi: 10.1038/s41586-021-04084-z PMC9298180 34759323

[98] Littlefield P Liu L Mysore V Shan Y Shaw DE Jura N. Structural analysis of the EGFR/HER3 heterodimer reveals the molecular basis for activating HER3 mutations. Sci Signal (2014) 7(354):ra114. doi: 10.1126/scisignal.2005786 25468994 PMC4492339

[99] Hashimoto Y Koyama K Kamai Y Hirotani K Ogitani Y Zembutsu A. A novel HER3-targeting antibody-drug conjugate, U3-1402, exhibits potent therapeutic efficacy through the delivery of cytotoxic payload by efficient internalization. Clin Cancer Res (2019) 25(23):7151–61. doi: 10.1158/1078-0432.CCR-19-1745 31471314

[100] Kiavue N Cabel L Melaabi S Bataillon G Callens C Lerebours F. ERBB3 mutations in cancer: biological aspects, prevalence and therapeutics. Oncogene (2020) 39(3):487–502. doi: 10.1038/s41388-019-1001-5 31519989

[101] Hafeez U Parslow AC Gan HK Scott AM. New insights into ErbB3 function and therapeutic targeting in cancer. Expert Rev Anticancer Ther (2020) 20(12):1057–74. doi: 10.1080/14737140.2020.1829485 32981377

[102] Ross JS Fakih M Ali SM Elvin JA Schrock AB Suh J. Targeting HER2 in colorectal cancer: The landscape of amplification and short variant mutations in ERBB2 and ERBB3. Cancer (2018) 124(7):1358–73. doi: 10.1002/cncr.31125 PMC5900732 29338072

[103] Engelman JA Zejnullahu K Mitsudomi T Song Y Hyland C Park JO. MET amplification leads to gefitinib resistance in lung cancer by activating ERBB3 signaling. Science (2007) 316(5827):1039–43. doi: 10.1126/science.1141478 17463250

[104] Yang X Chen Y Li M Zhu W. ERBB3 methylation and immune infiltration in tumor microenvironment of cervical cancer. Sci Rep (2022) 12(1):8112. doi: 10.1038/s41598-022-11415-1 35581263 PMC9114106

[105] Weickhardt AJ Lau DK Hodgson-Garms M Lavis A Jenkins LJ Vukelic N. Dual targeting of FGFR3 and ERBB3 enhances the efficacy of FGFR inhibitors in FGFR3 fusion-driven bladder cancer. BMC Cancer (2022) 22(1):478. doi: 10.1186/s12885-022-09478-4 35501832 PMC9063072

[106] Kang YY Liu Y Wang ML Guo M Wang Y Cheng ZF. Construction and analyses of the microRNA-target gene differential regulatory network in thyroid carcinoma. PloS One (2017) 12:e0178331. doi: 10.1371/journal.pone.0178331 28570571 PMC5453480

[107] Moore LD Le T Fan G. DNA methylation and its basic function. Neuropsychopharmacology (2013) 38(1):23–38. doi: 10.1038/npp.2012.112 22781841 PMC3521964

[108] Mattei AL Bailly N Meissner A. DNA methylation: a historical perspective. Trends Genet (2022) 38(7):676–707. doi: 10.1016/j.tig.2022.03.010 35504755

[109] Saghafinia S Mina M Riggi N Hanahan D Ciriello G. Pan-cancer landscape of aberrant DNA methylation across human tumors. Cell Rep (2018) 25(4):1066–80.e8. doi: 10.1016/j.celrep.2018.09.082 30355485

[110] He J Xu Q Jing Y Agani F Qian X Carpenter R. Reactive oxygen species regulate ERBB2 and ERBB3 expression via miR-199a/125b and DNA methylation. EMBO Rep (2012) 13(12):1116–22. doi: 10.1038/embor.2012.162 PMC3512405 23146892

[111] Zhong J Shan W Zuo Z. Norepinephrine inhibits migration and invasion of human glioblastoma cell cultures possibly via MMP-11 inhibition. Brain Res (2021) 1756:147280. doi: 10.1016/j.brainres.2021.147280 33515535 PMC7904089

[112] Evangelou AI Winter SF Huss WJ Bok RA Greenberg NM. Steroid hormones, polypeptide growth factors, hormone refractory prostate cancer, and the neuroendocrine phenotype. J Cell Biochem (2004) 91(4):671–83. doi: 10.1002/jcb.10771 14991759

[113] Slominski RM Raman C Chen JY Slominski AT. How cancer hijacks the body's homeostasis through the neuroendocrine system. Trends Neurosci (2023) 46(4):263–75. doi: 10.1016/j.tins.2023.01.003 PMC10038913 36803800

[114] Kosińska I Nitsch- Osuch A. [Benzo(a)pyrene in atmospheric and indoor air, health hazards and possibilities of limitation]. Pol Merkur Lekarski (2020) 49(286):282–8. 32827427

[115] Michałowicz J. Bisphenol A–sources, toxicity and biotransformation. Environ Toxicol Pharmacol (2014) 37(2):738–58. doi: 10.1016/j.etap.2014.02.003 24632011

[116] Scheiber I Dringen R Mercer JF. Copper: effects of deficiency and overload. Met Ions Life Sci (2013) 13:359–87. doi: 10.1007/978-94-007-7500-8_11 24470097

[117] Luine VN. Estradiol and cognitive function: past, present and future. Horm Behav (2014) 66(4):602–18. doi: 10.1016/j.yhbeh.2014.08.011 PMC4318702 25205317

[118] Shagufta Ahmad I. Tamoxifen a pioneering drug: An update on the therapeutic potential of tamoxifen derivatives. Eur J Med Chem (2018) 143:515–31. doi: 10.1016/j.ejmech.2017.11.056 29207335

[119] Snyder KR Sparano N Malinowski JM. Raloxifene hydrochloride. Am J Health Syst Pharm (2000) 57(18):1669–75; quiz 1676-8. doi: 10.1093/ajhp/57.18.1669 11006795

[120] Kaur T Madgulkar A Bhalekar M Asgaonkar K. Molecular docking in formulation and development. Curr Drug Discov Technol (2019) 16(1):30–9. doi: 10.2174/1570163815666180219112421 29468973

[121] Lee YT Tan YJ Oon CE. Molecular targeted therapy: Treating cancer with specificity. Eur J Pharmacol (2018) 834:188–96. doi: 10.1016/j.ejphar.2018.07.034 30031797

[122] Overington JP Al- Lazikani B Hopkins AL. How many drug targets are there. Nat Rev Drug Discov (2006) 5(12):993–6. doi: 10.1038/nrd2199 17139284

[123] Leary A Evans A Johnston SR A'Hern R Bliss JM Sahoo R. Antiproliferative effect of lapatinib in HER2-positive and HER2-negative/HER3-high breast cancer: results of the presurgical randomized MAPLE trial (CRUK E/06/039). Clin Cancer Res (2015) 21(13):2932–40. doi: 10.1158/1078-0432.CCR-14-1428 25398453

[124] Ohno K Shibata T Ito KI. Epidermal growth factor receptor activation confers resistance to lenvatinib in thyroid cancer cells. Cancer Sci (2022) 113:3193–210. doi: 10.1111/cas.15465 PMC9459297 35723021

[125] Cheng L Jin Y Liu M Ruan M Chen L. HER inhibitor promotes BRAF/MEK inhibitor-induced redifferentiation in papillary thyroid cancer harboring BRAFV600E. Oncotarget (2017) 8:19843–54. doi: 10.18632/oncotarget.15773 PMC5386727 28423638

[126] Lin S Lv Y Xu J Mao X Chen Z Lu W. Over-expression of Nav1.6 channels is associated with lymph node metastases in colorectal cancer. World J Surg Oncol (2019) 17(1):175. doi: 10.1186/s12957-019-1715-4 31672162 PMC6824047

[127] Hu J Xu J Li M Zhang Y Yi H Chen J. Targeting lymph node sinus macrophages to inhibit lymph node metastasis. Mol Ther Nucleic Acids (2019) 16:650–62. doi: 10.1016/j.omtn.2019.04.016 PMC6529739 31121477

[128] Chung EJ Lee SH Baek SH Park IS Cho SJ Rho YS. Pattern of cervical lymph node metastasis in medial wall pyriform sinus carcinoma. Laryngoscope (2014) 124(4):882–7. doi: 10.1002/lary.24299 23832757

[129] Lee SK Lee KW Kim S Choi MY Kim J Lee J. Lymph node metastasis in patients with frozen section analyses that are negative for tumors. Oncology (2012) 83(1):31–7. doi: 10.1159/000336486 22722529

[130] Holtkamp LH Wang S Wilmott JS Madore J Vilain R Thompson JF. Detailed pathological examination of completion node dissection specimens and outcome in melanoma patients with minimal (<0.1 mm) sentinel lymph node metastases. Ann Surg Oncol (2015) 22(9):2972–7. doi: 10.1245/s10434-015-4615-z 25990968

