# Supplementary figures and images for: Exploration and validation of key genes associated with early lymph node metastasis in thyroid carcinoma using weighted gene co-expression network analysis and machine learning
Source: Front Endocrinol (Lausanne). 2023 Dec 8;14:1247709. doi: 10.3389/fendo.2023.1247709 (PMC10739373; doi:10.3389/fendo.2023.1247709)

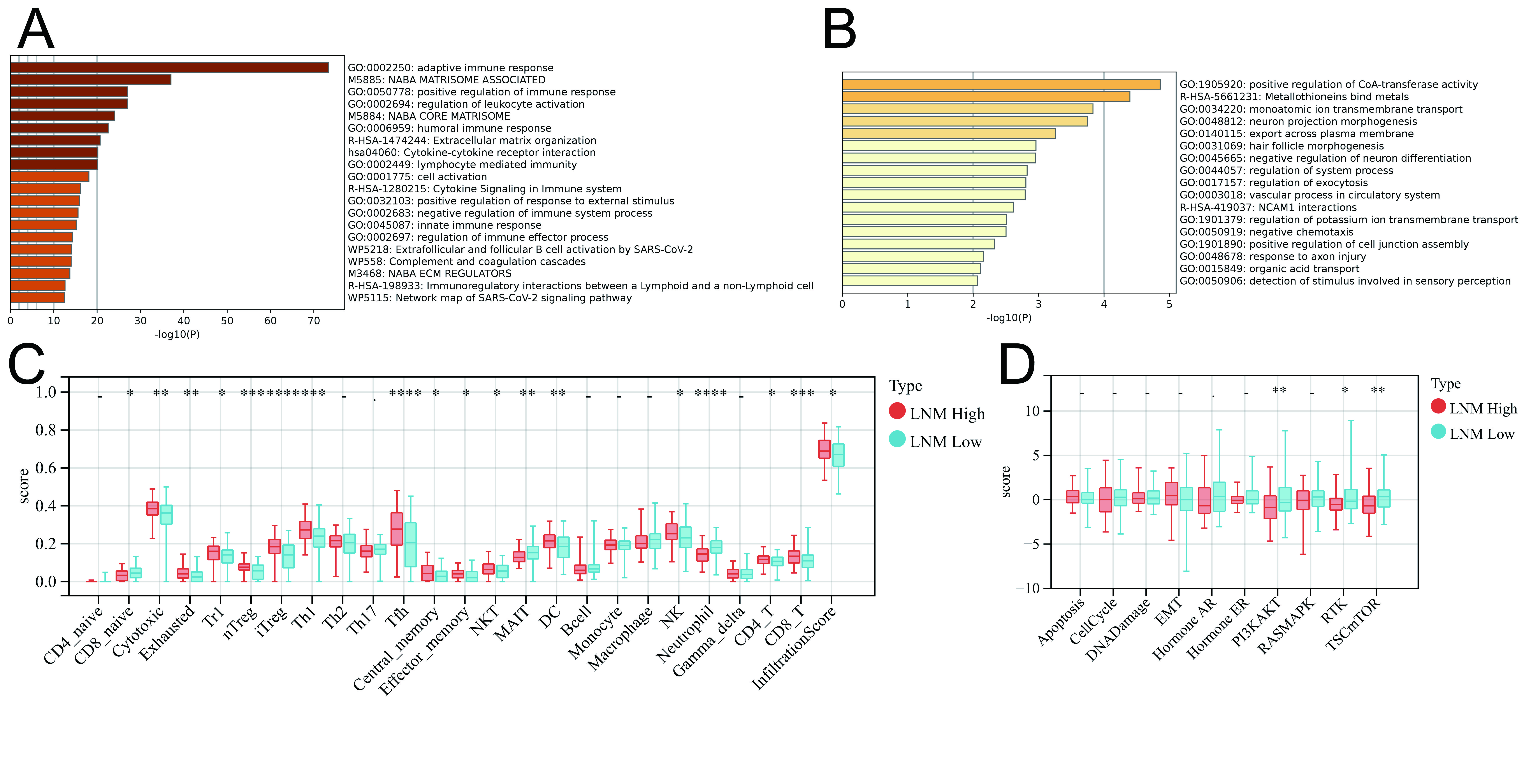

Supplement: Supplementary Figure 1 — Enrichment analysis results for up- (A) and down-regulated (B) genes in THCA. Distinctive patterns in immune infiltration (C) and cancer-related pathway activation (D) in THCA patients, stratified by high or low LNM potentials. [file Image_1.jpeg]

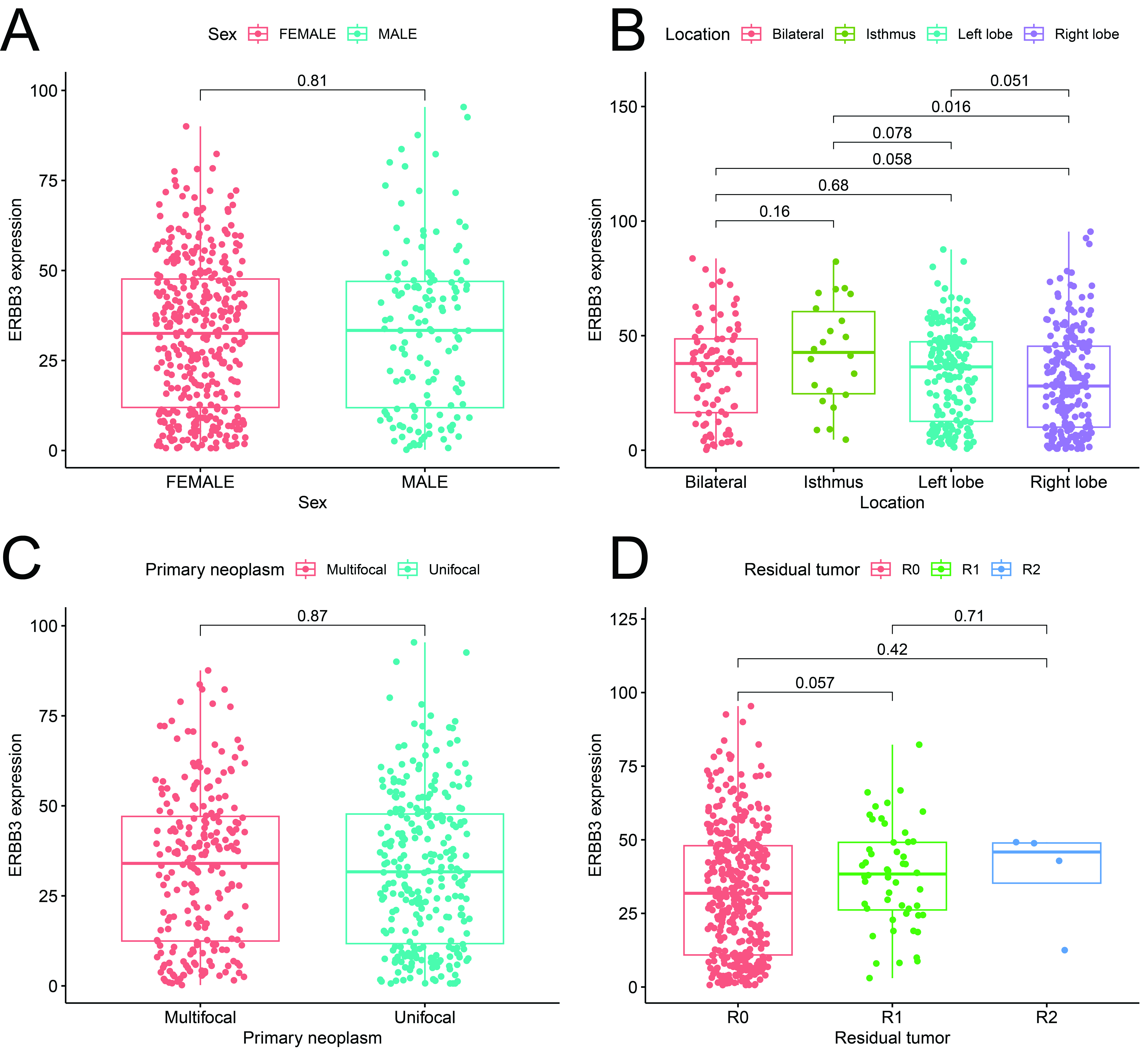

Supplement: Supplementary Figure 4 — Expression of ERBB3 in THCA patients differentiated by gender (A), site of occurrence (B), number of primary tumors (C), and extent of surgical resection (D). [file Image_4.jpeg]

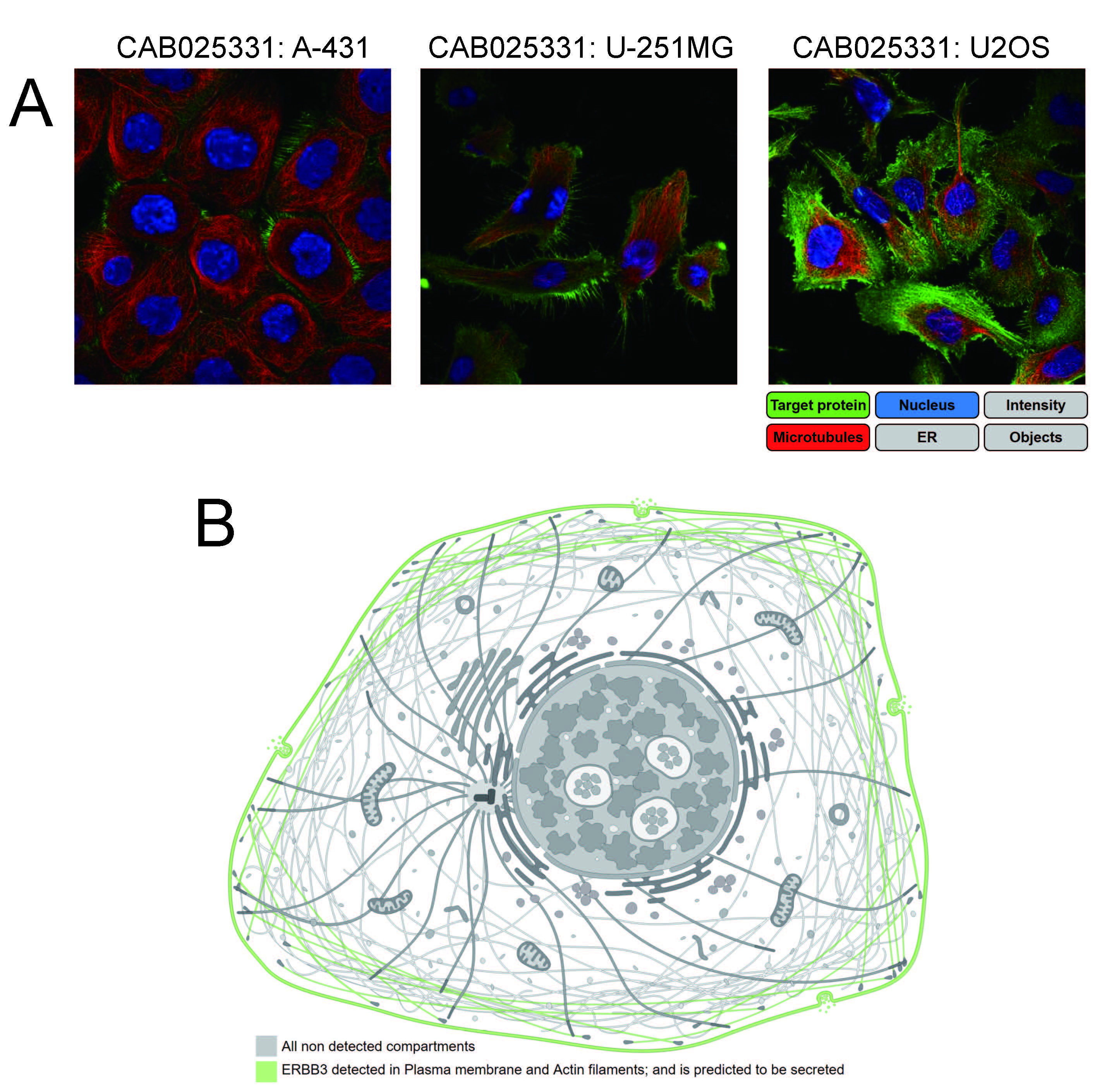

Supplement: Supplementary Figure 7 — (A) Subcellular localization of ERBB3 protein in various tumor cells. (B) Schematic representation of subcellular localization of ERBB3 protein in tumor cells. [file Image_7.jpeg]

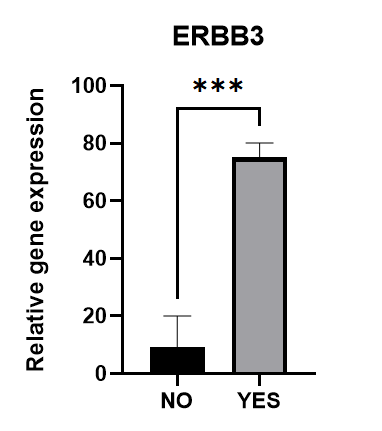

Supplement: Supplementary Figure 9 — qRT-PCR verification of expression of ERBB3 in THCA with (Yes) and without (No) lymph node metastasis (***: p<0.001). [file Image_9.png]
